# Multi-inch single-crystalline perovskite membrane for high-detectivity flexible photosensors

**DOI:** 10.1038/s41467-018-07440-2

**Published:** 2018-12-13

**Authors:** Yucheng Liu, Yunxia Zhang, Zhou Yang, Haochen Ye, Jiangshan Feng, Zhuo Xu, Xu Zhang, Rahim Munir, Jia Liu, Ping Zuo, Qingxian Li, Mingxin Hu, Lina Meng, Kang Wang, Detlef-M. Smilgies, Guangtao Zhao, Hua Xu, Zupei Yang, Aram Amassian, Jiawei Li, Kui Zhao, Shengzhong(Frank) Liu

**Affiliations:** 1 Key Laboratory of Applied Surface and Colloid Chemistry, Ministry of Education; Shaanxi Key Laboratory for Advanced Energy Devices; Shaanxi Engineering Lab for Advanced Energy Technology; Institute for Advanced Energy Materials; School of Materials Science and Engineering, Shaanxi Normal University, 710119 Xi’an, P. R. China; 2 iChEM, Dalian National Laboratory for Clean Energy, Dalian Institute of Chemical Physics, Chinese Academy of Sciences, 116023 Dalian, P. R. China; 3 Division of Physical Sciences and Engineering, Solar and Photovoltaics Engineering Center, King Abdullah University of Science and Technology (KAUST), Thuwal, 23955-6900 Saudi Arabia; 4 Cornell High Energy Synchrotron Source, Cornell University, Ithaca, NY 14850 USA; 5 School of Physics and Information Technology, Shaanxi Normal University, 710119 Xi’an, P. R. China

## Abstract

Single crystalline perovskites exhibit high optical absorption, long carrier lifetime, large carrier mobility, low trap-state-density and high defect tolerance. Unfortunately, all single crystalline perovskites attained so far are limited to bulk single crystals and small area wafers. As such, it is impossible to design highly demanded flexible single-crystalline electronics and wearable devices including displays, touch sensing devices, transistors, etc. Herein we report a method of induced peripheral crystallization to prepare large area flexible single-crystalline membrane (SCM) of phenylethylamine lead iodide (C_6_H_5_C_2_H_4_NH_3_)_2_PbI_4_ with area exceeding 2500 mm^2^ and thinness as little as 0.6 μm. The ultrathin flexible SCM exhibits ultralow defect density, superior uniformity and long-term stability. Using the superior ultrathin membrane, a series of flexible photosensors were designed and fabricated to exhibit very high external quantum efficiency of 26530%, responsivity of 98.17 A W^−1^ and detectivity as much as 1.62 × 10^15^ cm Hz^1/2^ W^−1^ (Jones).

## Abstract (web summary)

Hybrid halide perovskite single crystals show excellent optoelectronic properties but their small size and large thickness limit their application. Herein Liu et al. grow large area ultrathin flexible crystalline membrane of layered perovskite and demonstrate high detectivity in the flexible photosensors.

## Introduction

Single-crystalline organic–halide-based perovskites have attracted tremendous research in the past few years due to their superior optoelectronic properties, including high optical absorption^1–3^, long carrier lifetime as much as >260 μs^4^, large carrier mobility (approximately 25–100 cm^2^ V^−1^ s^−1^) comparing to other organic semiconductors^3,5–7^, trap-state-density measured as low as 10^9^–10^10^ cm^−3^^3,5–7^, and high-exciton diffusion length reported as long as 1 mm^7,8^. To date, perovskite single crystals reported^9^ have included MAPbX_3_ (MA = CH_3_NH_3_, X = Cl, Br, I)^3,5–7,10^, MACaI_3_^11^, MASnX_3_^12,13^, FAPbX_3_ (FA = HC(NH_2_)_2_, X = Cl, Br, I)^2,14–16^, FASnX_3_^12,17^, NH(CH_3_)_3_SnX_3_^17^, Cs_2_PdBr_6_^18^, Cs_2_AgBiBX_6_^19,20^, CsPbX_3_^21–24^, and mixed ion halide perovskites^25–29^. Unfortunately, all crystalline perovskite materials available at present are limited to bulk single crystals and wafers with thickness much larger than sub-millimeters while their lateral dimensions limited to only sub-centimeter beyond a few large crystals reported by our group^1–3,30^. As such, it is impossible to use the bulk single crystals to design highly demanded flexible electronics and wearable devices, such as displays, touch sensing devices, transistors, photosensors, and solar cells. Even though the microcrystalline perovskite thin films may be used in flexible electronic devices, their efficiencies are fairly low^31–33^. Beyond efforts in our group with limited success^1,2,30^, only a couple of papers reported on thin perovskite single crystals. Specifically, a cavitation-triggered asymmetric crystallization strategy has been developed to form approximately 2.3 × 1.8 mm^2^ sized MAPbBr_3_ single-crystalline sheets^34^. The space confined condition has been introduced to grow 6 × 8 mm^2^, 5 × 4 mm^2^, and approximately 2.8 × 3.9 mm^2^ perovskite crystalline thin films^35–37^. However, they are too small to be usable for real device applications. Most recently, a 120 cm^2^ (approximately 18.5 × 6.5 cm^2^) films MAPbBr_3_ was grown, but it is too thick (0.4 mm) to be flexible^38^. It appears to be indeed intrinsically challenged to grow flexible perovskite single-crystalline membrane (SCM).

Recently, two-dimensional (2D) layered lead halide perovskite has come to the spotlight for its superior environmental stability. In fact, it is recognized as a promising candidate for potential commercial applications in solar cells, light-emitting diodes, and other optoelectronics^39–50^. More recently, 2D layered lead halide perovskite, particularly (C_6_H_5_C_2_H_4_NH_3_)_2_PbX_4_ ((PEA)_2_PbX_4_, X = Cl, Br, I) compounds, have been rediscovered for their astonishing stability and potential high solar cell efficiency^42,51,52^. However, up to now all research has been limited to the microcrystalline thin films (MCTFs) and nanocrystalline materials. To take full advantage of its structural, optical, and electronic properties, it is imperative to prepare single crystals. Even though a few limited 2D layered (PEA)_2_PbI_4_ single-crystalline perovskites have already been prepared, unfortunately, none of them is large enough for large-area device applications. Moreover, large-area SCM is needed in order to develop larger-scale integrated optoelectronic devices. In fact, so far there are only three reports on the (PEA)_2_PbI_4_ single crystals, with dimensions limited to approximately 0.2 × 0.2, 2 × 1, and 8.7 × 3.1 mm^2^, respectively^53–55^. It is apparent that the inherent nature of random growth to all directions makes it challenging to control membrane thickness while at the meantime large lateral size is demanded.

In this paper, we report a “induced peripheral crystallization” (IPC) method to grow large-area SCM of (C_6_H_5_C_2_H_4_NH_3_)_2_PbI_4_ ((PEA)_2_PbI_4_ SCM) with well-controlled thinness to approximately 0.6 µm, while its area expanded to >2500 mm^2^ (73 × 35 mm^2^), much larger than what is reported in literature to the best of our knowledge^54,55^. It is found that the ultrathin single-crystalline perovskite membrane displays ultralow defect density, long-term stability, and excellent flexibility. Meanwhile, we have designed and fabricated flexible photosensors using the ultrathin large (PEA)_2_PbI_4_ SCM. It is found that the present photosensor shows very high external quantum efficiency (EQE) as much as 26,530%, responsivity (*R*) as high as 98.17 A W^−1^, and detectivity (D*) as large as 1.62 × 10^15^ cm Hz^1/2^ W^−1^ (Jones). It should be noted that this detectivity value is not only among the highest detectivity reported to date for all single-crystalline and thin-film perovskite photosensors^56^, it is even higher than devices based on the state-of-the-art material including single-crystalline silicon^56^, InGaAs^57^, GaAs^58^, CdTe^59^, and GaN^60^. Moreover, the flexible membrane photosensor shows excellent mechanical stability.

## Results

### SCM growth

There have been two typical methods developed to grow the hybrid lead halide perovskite CH_3_NH_3_PbI_3_ single crystals. The first uses hydrogen iodide acid as the solvent in which the solubility of perovskite increases as temperature rises. By slowly lowering the temperature of the saturated solution, the crystals are harvested^7,61^. In comparison, the second one uses γ-butyrolactone (GBL) as the solvent in which solubility of the perovskite decreases as temperature rises. The crystals were collected by first preparing a saturated solution and then slowly increases its temperature to enter into the oversaturation zone^1–3^.

There are a few key parameters that are known to affect crystal quality and its growth process. In general, the lower the growth rate, the higher the crystalline quality is. The crystal growth is directly controlled by two processes: the first is solute diffusion from the solution to the crystallite surface, and the second is the solute deposition onto the crystallite. The former can be well controlled by regulating solution temperature for the diffusion rate that is well known to change exponentially with the temperature^62^. However, it is difficult to control the solute deposition process. We therefore maintain the process well under the diffusion-controlled region and use relatively low temperature to regulate the diffusion rate, hence the crystal growth rate and quality. On the other hand, it is key to control the growth along the solubility curve for, when it is in the well-oversaturated region, small crystallites form instantly with plenty of defects. When it goes into the unsaturated region, the crystal formed may start to be dissolved into the solution. Only when the solute concentration is controlled just over the solubility curve or slightly oversaturated, the crystallization kinetics allows high-quality crystal growth on the already formed crystallite or seed with no extraneous nucleation^63^.

**Fig. 1:**
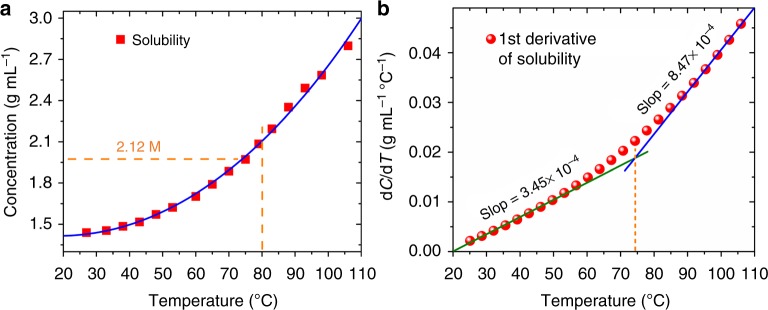
Temperature-dependent solubility of (PEA)_2_PbI_4_. **a** Solubility of (PEA)_2_PbI_4_ SCM in GBL as a function of temperature. **b** Solubility derivative with respect to temperature for (PEA)_2_PbI_4_ SCM in GBL

More quantitatively, crystal growth rate is directly proportional to the first-order derivative of solubility and the temperature ramp rate according to the oversaturation model^64^:

1 $$\frac{{{\mathrm{d}}m}}{{{\mathrm{d}}t}} = - \frac{1}{2}V \cdot \frac{{{\mathrm{d}}C(T)}}{{{\mathrm{d}}T}}\frac{{{\mathrm{d}}T}}{{{\mathrm{d}}t}}$$

where *m* is the mass of the crystal, *C* the solution concentration, and *V* the solution volume that changes very little during the entire crystallization process, hence can be treated as a constant. Figure 1a shows the solubility of (PEA)_2_PbI_4_ perovskite in the GBL solvent measured as a function of temperature. It is clear that, in the temperature range of 27–106 °C, the solubility of (PEA)_2_PbI_4_ increases with temperature. Based on the solubility data measured in Fig. 1a, its first-order derivative with respect to temperature is calculated and plotted in Fig. 1b. As the solution temperature can be well controlled over time, so is the temperature ramp rate. Once the temperature is set, the solubility derivative is defined based on the curve in Fig. 1b.

To grow single crystals along lateral dimensions while limiting its growth in thickness, it is critical to selectively control its growth rate along different directions. It is found that the best temperature range is 25–80 °C in which the solubility derivative is about 3.45 × 10^−4^, apparently lower comparing to its value 8.47 × 10^−4^ at higher temperature beyond 80 °C, as shown in Fig. 1b. For the best quality at acceptable growth rate, we further regulated the temperature ramp rate to 1 °C h^−1^.

**Fig. 2:**
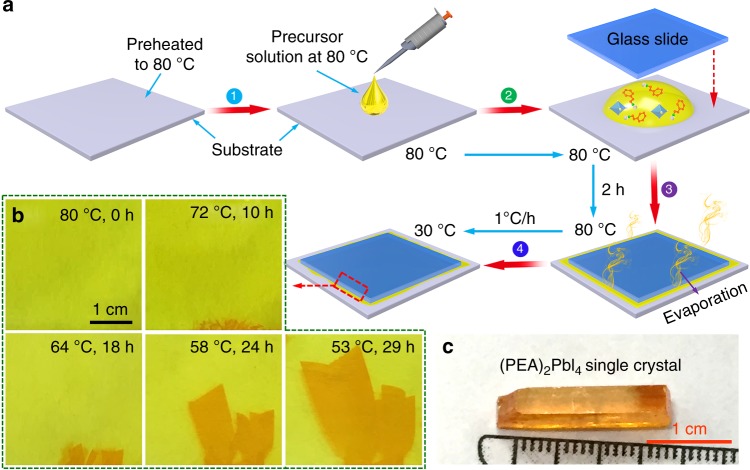
IPC growth process of (PEA)_2_PbI_4_ SCM. **a** Schematic illustration of the IPC procedure to grow 2D layered (PEA)_2_PbI_4_ SCM. **b** Photos of a (PEA)_2_PbI_4_ SCM taken at different stages of the growth process. **c** Photo of a typical (PEA)_2_PbI_4_ crystal.

Figure 2a illustrates the IPC growth procedure. Briefly, PbI_2_ and C_6_H_5_C_2_H_4_NH_3_I (1:2 molar ratio) were dissolved in γ-butyrolactone (GBL) at 80 °C under active mixing for 24 h to generate the (PEA)_2_PbI_4_ precursor solution (2.12 M). The solution was then filtered prior to crystal growth. For the growth of the (PEA)_2_PbI_4_ SCM, a drop of the prepared precursor solution was pipetted onto a glass slide substrate (or flexible plastic substrate, polyethylene terephthalate (PET) substrate) preheated to 80 °C as shown in step 1. Then, a second glass slide with smaller lateral dimensions was placed onto the precursor solution drop. The solution was squeezed to spread evenly between two slides (step 2). It should be noted that part of the precursor solution was squeezed out to the edge of the top slide. Step 3 is to maintain the temperature at 80 °C in an enclosed oven. In such case, the evaporation of the precursor solution along the edge of the top slide was well controlled at a consistent rate. In about 2 h, nucleation and crystallization occurred at the edge of the top slide, as evidenced by some shinny crystallites. Further gradient decrease of the temperature from 80 to 30 °C at a rate of 1 °C h^−1^ led to the growth of the (PEA)_2_PbI_4_ SCM between slides (step 4). In order to avoid crystallite growth in solution, the precursor was prepared at 80 °C with concentration controlled at 2.12 M (PEA)_2_PbI_4_ in GBA to warrant an unsaturated solution free of particulates that may serve as nucleation seeds. Everything used in the process, including glass slides and pipet, were preheated to 80 °C to avoid local temperature change and associated crystallization during the entire operation of steps 1–3. At this temperature, the solution (2.12 M) is unsaturated, therefore there is no possibility of crystallite formation anywhere. As the solvent around edges of the second glass slide was evaporated, the local solution concentration was slowly increased. When it surpasses the solubility curve, small crystallites start to form and grow larger. In step 4, temperature of the set-up was gradually lowered. When it was dropped to below 75 °C, the solution became oversaturated, leading to crystal growth surrounding the nucleation seeds. As the process continues, the crystals grow into the slit until they filled the entire space. It is of vital importance for the edges of the second plate to provide nucleation sites to direct the crystals to grow inwards into the slit, rather than to form nanocrystalline powder from the solution. Figure 2b shows five photographs of the (PEA)_2_PbI_4_ SCMs completed at different stages of the growth process. It shows that, without any seed crystal in the slit, there was no crystal formed when the temperature was kept at 80 °C. This is expected for, at the 80 °C, the solution is unsaturated. As the temperature was dropped to 72 °C, small crystallites were formed along the edge of the glass slide. When the temperature was dropped to 64 °C, the small crystallite grew larger and eventually forming 5 × 3 mm^2^ SCM in the slit. When the temperature was further lowered to 53 °C, the SCM grew larger to a 26 × 15 mm^2^ SCM. Apparently, the perovskite crystallites are formed from the edge of the second slide, they then grow larger into the slit until the space is filled. However, when the identical procedure was carried out in a closed petri dish without using any slide from 80 to 30 °C, there formed a few well-shaped single crystals with habitant rectangular parallelepiped, as shown in Fig. 2c.

### Thickness control and flexibility

**Fig. 3:**
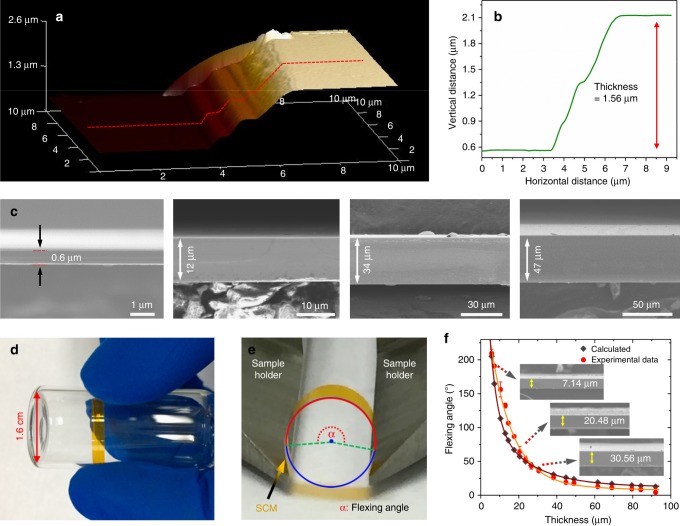
Thickness control and flexibility of (PEA)_2_PbI_4_ SCMs. **a** 3D AFM image of a (PEA)_2_PbI_4_ SCM and **b** height profile of the line scan. **c** Cross-sectional SEM images of the (PEA)_2_PbI_4_ SCMs with different thicknesses (0.6, 12, 34, 47 μm). **d** Photo of a piece of (PEA)_2_PbI_4_ SCM, 3.5 μm in thickness, wrapped around a small tube (1.6 cm in diameter) to show its flexibility. **e** A photo showing the flexing angle measurement for the (PEA)_2_PbI_4_ SCM. **f** Correlation of the (PEA)_2_PbI_4_ SCM thickness and corresponding flexing angle, with the insets showing cross-sectional SEM images of the (PEA)_2_PbI_4_ SCMs with three thicknesses (7.14, 20.48, 30.56 μm)

Figure 3a, b and Supplementary Fig. 1a, b present three-dimensional (3D) atomic force microscopy (AFM) images, with the height profiles clearly revealing successful fabrication of (PEA)_2_PbI_4_ SCM in thickness of 1.56 and 1.21 μm, respectively. It is found that the thickness of the (PEA)_2_PbI_4_ SCMs can be controlled by adjusting the cooling rate. The thickness distribution of the (PEA)_2_PbI_4_ SCMs obtained with various cooling rates are as shown in Supplementary Fig. 2. Figure 3c provides cross-sectional scanning electron microscopic (SEM) images of a few typical (PEA)_2_PbI_4_ SCMs prepared using the IPC method with thickness ranging from 0.6 to 50 μm, with detailed characterizations and analyses shown in Supplementary Fig. 3. Supplementary Fig. 3a, b presents photos and SEM images of a representative (PEA)_2_PbI_4_ SCM, there is no cracking found in the area of 4.1 × 1.8 mm^2^ inspected. Careful examination at higher resolution (Supplementary Fig. 3c) further confirms that the SCM is indeed single crystalline without observable grain boundaries. Supplementary Fig. 3d, e provides analysis using the energy-dispersive X-ray spectroscopy (EDS). It shows that the atomic ratio: C:Pb:I is approximately 18:1:4, consistent with the stoichiometry of (C_6_H_5_C_2_H_4_NH_3_)_2_PbI_4_. In addition, EDS mapping analysis (Supplementary Fig. 3f–i) over area 0.56 × 0.88 mm^2^ shows very uniform atomic distribution. Moreover, there are some terraces observed along the edge of the (PEA)_2_PbI_4_ SCMs (Supplementary Fig. 4), demonstrating that the (PEA)_2_PbI_4_ SCM is indeed a layered (2D) material. It is worthwhile to note that the 2D layered perovskite (PEA)_2_PbI_4_ SCMs are flexible. As shown in Fig. 3d, the (PEA)_2_PbI_4_ SCM with thickness of 3.5 μm can be wrapped around on a small tube (1.6 cm in diameter) without any deformation, demonstrating that the (PEA)_2_PbI_4_ SCM is of very good flexibility. In fact, the (PEA)_2_PbI_4_ SCM can be flexed in a wide range without observable fracture. Figure 3e illustrates the measurement procedure for the flexing angle, the maximum bending angle tolerated before fracture, and Supplementary Fig. 5 shows a test for a 7.14-μm-thick membrane in the range of flexing angle from 45° to 195°. Figure 3f shows the flexing angle as a function of the (PEA)_2_PbI_4_ SCM thicknesses. It is found that, the thinner the membrane, the larger the flexing angle, the more flexible it is. Moreover, the curve showed a biexponential feature, consistent with the calculated result from elastic flexing deformation model for the layered materials (details described in Supplementary Fig. 6)^65^.

2 $$\alpha = \frac{{180^oL}}{{\pi d}}$$

where *L* is the maximum stretching length, *α* is the flexing angle, and *d* is half the thickness of the (PEA)_2_PbI_4_ SCM.

The above results demonstrate that the IPC is indeed a highly effective method to fabricate large-area flexible (PEA)_2_PbI_4_ SCM. More importantly, flexible PET substrate has also been successfully tested to grow the (PEA)_2_PbI_4_ SCM, as the samples shown in Supplementary Fig. 7. Compared to the brittle 3D perovskite single crystals, e.g., MAPbX_3_ and FAPbI_3_ as we reported earlier^1–3,30^, the 2D flexible layered (PEA)_2_PbI_4_ SCM is of obvious advantages in flexible electronic devices, including flexible displays, wearable electronics, portable devices, ultrathin transistors, and special sensors.

### **S**tructural characterization

**Fig. 4:**
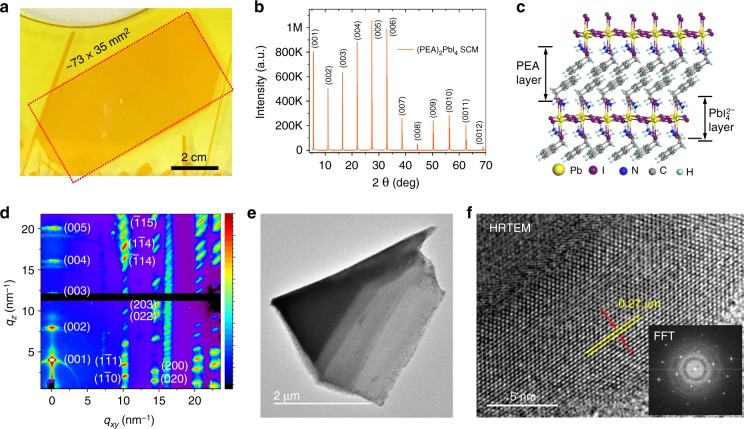
Structural of (PEA)_2_PbI_4_ SCM. **a** Photo of the (PEA)_2_PbI_4_ SCM sample (73 × 35 mm^2^) used for XRD measurements. **b** XRD pattern of the (PEA)_2_PbI_4_ SCM. **c** Layered crystal structure of the (PEA)_2_PbI_4_. **d** GIWAXS image of the 2D layered (PEA)_2_PbI_4_ SCM. **e**, **f** TEM image and HRTEM image of a (PEA)_2_PbI_4_ SCM, with the d-spacing measured assigned to the (006) lattice plane

X-ray diffraction (XRD), grazing-incidence wide-angle X-ray scattering (GIWAXS), and transmission electron microscopy (TEM) have been used to characterize the (PEA)_2_PbI_4_ SCM samples. Figure 4a shows a photo of a typical (PEA)_2_PbI_4_ SCM (73 × 35 mm^2^) on glass substrate used for the XRD measurement. The XRD in Fig. 4b shows a series of sharp, intense, and well-defined diffraction peaks repeating periodically, signature of the (00*h*) (*h* = 1, 2, 3…) planes in layered (PEA)_2_PbI_4_ crystalline structure^54,55^ as shown in Fig. 4c. As it was previously observed, the 2D plane of the (PEA)_2_PbI_4_ SCM are preferentially parallel to the substrate^45,55^. Supplementary Fig. 8 shows the rocking curve corresponding to the (005) plane with the full-width at half maximum (FWHM) only 0.1055°, demonstrating respectable crystalline quality. To gain more insights into the orientation, GIWAXS were performed on both the (PEA)_2_PbI_4_ SCM and a sample of corresponding MCTF. The GIWAXS image and the intensity versus *q* for the scattering features of the (PEA)_2_PbI_4_ SCM are shown in Fig. 4d and Supplementary Fig. 9, respectively. We observed sharp, discrete Bragg spots at *q* values of 10.14, 14.40, 16.16, 20.13, and 22.66 nm^−1^, corresponding to the (00*h*) (*h* = 1, 2, 3…) planes in layered (PEA)_2_PbI_4_ crystalline structure. This indicates perfect ordering and closed packing between each crystalline slab. Meanwhile, the synchrotron diffraction data indicate that the major perovskite growth is confined within the (00*h*) plane that is perpendicular to the *q*_*z*_ direction^66^. In contrast, strong diffraction rings at *q* = 18.8 nm^−1^ and *q* *=* 23.8 nm^−1^ are observed for the microcrystalline counterpart (Supplementary Fig. 10), indicating considerable randomness in the orientation of the crystal grains due to the discontinued/misaligned stacking. The (PEA)_2_PbI_4_ SCM was further studied using high-resolution TEM analysis (HRTEM). Figure 4e shows the TEM image and Fig. 4f shows the crystal lattice image of the (PEA)_2_PbI_4_ SCM with a lattice spacing of 0.27 nm, corresponding to the perovskite (006) plane, consistent with the XRD result.

### Optical and transport properties

**Fig. 5:**
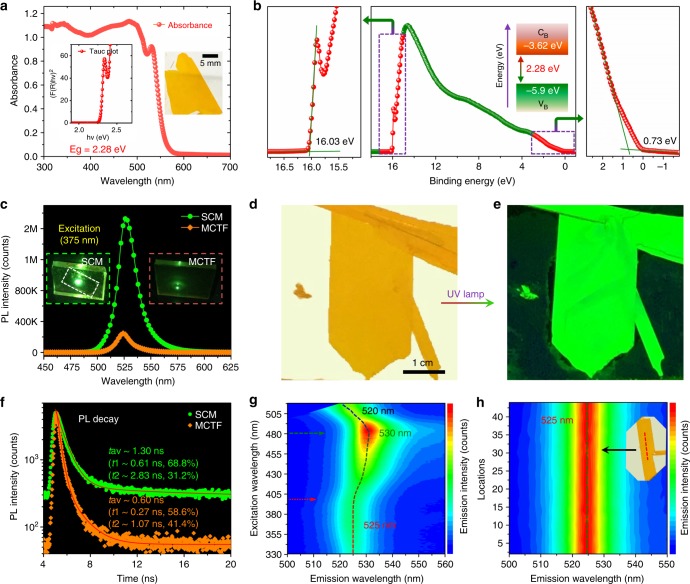
Optical properties of (PEA)_2_PbI_4_ SCM. **a** The absorbance spectrum and corresponding Tauc plot of the (PEA)_2_PbI_4_ SCM showing a band gap 2.27 eV. The inset shows the photograph of the (PEA)_2_PbI_4_ SCM used for absorbance measurement. **b** Ultraviolet photoemission spectroscopy (UPS) of the (PEA)_2_PbI_4_ SCM. Insets: energy band diagram for the (PEA)_2_PbI_4_ SCM. **c** Photoluminescence spectrum of the (PEA)_2_PbI_4_ SCM and MCTF excited at 375 nm. Inset: the photo of the (PEA)_2_PbI_4_ SCM and MCTF excited at 375 nm. **d**, **e** Photographs of the (PEA)_2_PbI_4_ SCM under weak room light and strong UV lamp. **f** Time-resolved PL of the (PEA)_2_PbI_4_ SCM and MCTF at 525 nm. The excitation laser beam wavelength is 375 nm. Inset: Photograph of the green photoluminescence of a (PEA)_2_PbI_4_ SCM (left) and MCTF (right) excited by a 375 nm laser beam. **g** 3D excitation–emission plot for the (PEA)_2_PbI_4_ SCM. Emission intensity rises with the color changing from blue to green and red. **h** Evolution of the photoluminescence spectrum shown using pseudocolor plot, indicating consistent emission peak wavelength and intensity across the large area

Optical properties of the (PEA)_2_PbI_4_ SCM were thoroughly studied, with the absorption spectrum showing a sharp absorption edge at 550 nm, corresponding to the bandgap of 2.28 eV (Fig. 5a). This phenomenon is consistent with the absorption curve reported independently by other group on bulk crystals^54^. The optical band gaps estimated from the corresponding Tauc plots show values of 2.28 and 2.35 eV for the (PEA)_2_PbI_4_ SCM and MCTF (Supplementary Fig. 11), respectively, in good agreement with previous report^53,54^. From ultraviolet photoelectron spectroscopy (UPS) results, we estimated the valence band edge position of (PEA)_2_PbI_4_ SCM to be −5.90 eV and for MCTF at −5.44 eV, relative to the vacuum level (Fig. 5b and Supplementary Fig. 12). The (PEA)_2_PbI_4_ SCM and MCTF samples were further characterized by photoluminescence (PL) spectra with a 375 nm laser excitation, as shown in Fig. 5c. The sharp and narrow peak at 525 nm (FWHM = 15.1 nm) demonstrates the high color purity (narrow emission bandwidth) of the (PEA)_2_PbI_4_ SCM, an attractive feature for light-emitting applications. The PL peak centered at 521 nm for the MCTF (PEA)_2_PbI_4_ is slightly blue-shifted compared to that in the (PEA)_2_PbI_4_ SCM located at 525 nm, indicating that the latter is indeed the SCM with lower defect density^67^. In addition, the PL intensity of the (PEA)_2_PbI_4_ SCM is much higher than that of the (PEA)_2_PbI_4_ MCTF under same test condition, as shown in the inset photograph. Figure 5d, e shows photos of the (PEA)_2_PbI_4_ SCM taken under weak room light and ultraviolet (UV) lamp, respectively. It shows that, under the weak white light, the membrane appears to be yellowish in color and very uniform across the surface area. When it is exposed to strong UV irradiation, it becomes bright green with very uniform appearance. Apparently, the UV lamp has effectively excited the PL peaked at 525 nm. The PL lifetimes of the (PEA)_2_PbI_4_ SCM and MCTF were measured using time-resolved PL. As shown in Fig. 5f, the decay curves show a biexponential feature with average lifetime of 1.3 and 0.6 ns, respectively. More interestingly, we found that the (PEA)_2_PbI_4_ SCM shows characteristic excitation-dependent fluorescence behavior, with the observed emission peaks shifting from 525 to 530 nm and to 520 nm with excitation wavelengths as shown in Fig. 5g. Specifically, when the excitation wavelength is <400 nm, the emission peak position is at 525 nm. When the excitation wavelength is changed from 400 to 480 nm, the emission peak shifts from 525 to 530 nm. Further increasing the excitation wavelength, the emission peak is shifted from 530 to 520 nm. This characteristic feature can also be observed on the MCTFs (Supplementary Fig. 13).

The PL is also used to study uniformity of the (PEA)_2_PbI_4_ SCM sample. Surprisingly, the PL emission peak from 44 different points taken across the SCM is sharply centered at 525 nm, with FWHM distributed within 14.8 ± 0.14 nm. More surprisingly, their peak intensity is maintained essentially the same at 3.87 ± 0.02 × 10^4^ counts (Fig. 5h and Supplementary Table 1). The standard deviation for these 44 tests is as low as 0.53% of their average intensity and only 0.95% for the FWHM, demonstrating good uniformity over large area for the high quality (PEA)_2_PbI_4_ SCM. The automated PL intensity and lifetime mapping performed over surface area of 1 cm^2^ also exhibits far superior uniformity comparing to the corresponding MCTF (PEA)_2_PbI_4_ sample (Supplementary Fig. 14). The excellent optical uniformity makes it a promising candidate for large-area optoelectronic applications.

Trap-state density is another key figure-of-merit for optoelectronic and photovoltaic applications. Both electron-only and hole-only devices were therefore designed and fabricated for the measurement using the space-charge-limited current (SCLC) method^3^. The dielectric constants (*ε*) was determined to be 5.8 ± 0.1 from the capacitance–frequency measurement in the range of 100 kHz to 30 MHz by impedance analyzer (Supplementary Fig. 15). Supplementary Fig. 16 presents the dark current–voltage (*I*–*V*) curves for the single-carrier devices using the (PEA)_2_PbI_4_ SCM. Based on 8 measurements, the hole-trap density (*n*_trap_) was calculated as (8.18 ± 2.67) × 10^10^ cm^-3^ (Supplementary Table 2), and the electron-trap density was calculated as (1.66 ± 0.88) × 10^11^ cm^−^^3^ (Supplementary Table 3). With low defect density and uniformity, the (PEA)_2_PbI_4_ SCM should be ideal for optoelectronic applications. For real-world usage, stability becomes a critical factor for consideration. The thermal stability of the (PEA)_2_PbI_4_ SCM was evaluated using thermogravimetric analysis (TGA), as shown in Supplementary Fig. 17. It shows that the (PEA)_2_PbI_4_ SCM does not exhibit any observable degradation until 230 °C. We also tested its stability in high humidity environment by subjecting it to approximately 65% relative humidity at 25 °C for 32 days. For the stability measurement, XRD was collected on a daily basis during the test period. As shown in Supplementary Fig. 18, there is no detectable decomposition in the XRD patterns after the long-term exposure, which suggests that the (PEA)_2_PbI_4_ SCM is highly stable in humid condition. As its MCTF counterpart decomposes after 24 h in the same test, it is believed that the better stability of SCM is attributed to essentially eliminated solvent residues, grain boundaries, inclusions, and voids within the crystal. Supplementary Fig. 19 shows water contact angle tests to compare SCM with MCTF. While the contact angle for the reference MCTF sample measured is only 76°, it is increased to as much as 94° for the (PEA)_2_PbI_4_ SCM sample, demonstrating that the (PEA)_2_PbI_4_ SCM is significantly poorer in wettability or better in water/moisture resistance.

### Device performance of flexible photosensor

**Fig. 6:**
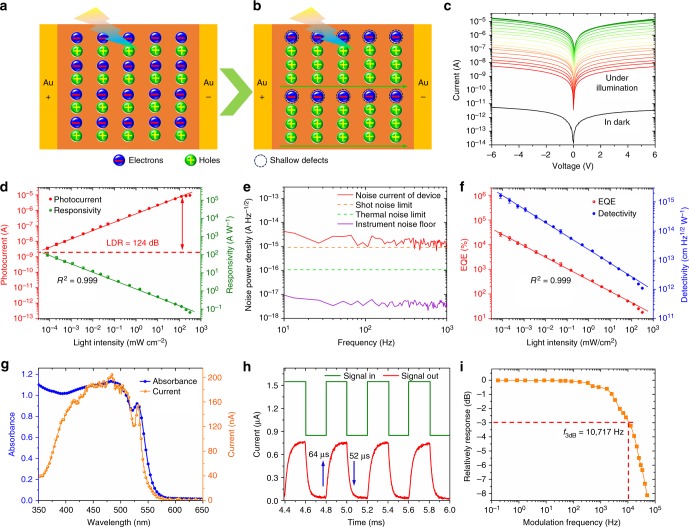
Performance of flexible (PEA)_2_PbI_4_ SCM photosensor. **a**, **b** Schematic illustrations of the photoelectric process and photoconductivity gain in the present Au/(PEA)_2_PbI_4_ SCM/Au device under light illumination. **c** The current–voltage (*I*–*V*) curves of the (PEA)_2_PbI_4_ SCM device measured in dark and under 460 nm wavelength illumination with various light intensities. **d** Photocurrent and responsivity for the (PEA)_2_PbI_4_ SCM photosensors within the incident light power density ranging from 8 × 10^−^^5^–310 mW cm^−^^2^ at wavelength 460 nm under a fixed 4 V bias. **e** Measured dark current noise at various frequencies of the (PEA)_2_PbI_4_ SCM photosensor with a 4 V bias. The measured instrument noise floor, calculated shot noise, and thermal noise limit are also included for reference. **f** EQE and D* for the (PEA)_2_PbI_4_ SCM photosensor within the incident light power density ranging from 8 × 10^−5^–310 mW cm^−^^2^ at wavelength 460 nm under a fixed 4 V bias. The points correspond to average values of measurements on 20 (PEA)_2_PbI_4_ SCM photosensors, and the error bars represent the standard deviation. **g** Absorbance spectrum of the (PEA)_2_PbI_4_ SCM and photoresponse spectrum of the photosensor illuminated using monochromatic light with wavelength ranging from 350 to 650 nm at 4 V bias. **h** Temporal photocurrent response of the (PEA)_2_PbI_4_ SCM photosensor. **i** Frequency response of the (PEA)_2_PbI_4_ SCM photosensor

With all above superior properties including low defect density, good uniformity, long-term stability and adequate flexibility, we are inspired to expand its application into flexible photosensors, as designed with the architecture schematically illustrated in Supplementary Fig. 20 and the working mechanism is described in Fig. 6a, b. Please note that the devices in this manuscript are designed using the metal–semiconductor–metal structure with interdigitated electrodes. As the photogenerated electrons may be trapped by shallow defects, holes may traverse between the electrodes for multiple times before recombination, in other words, holes drift along the electric field until they reach the electrode while electrons remain trapped in “doping centers.” To maintain charge neutrality, mobile holes are continuously recirculated between electrodes (majority carrier recirculation)^68,69^. Consequently, for each electron–hole pair created, holes may bounce between two electrodes repeatedly for multiple times, resulting in recirculation for abnormally high photoconductive gain.

The effectively illuminated device area is measured to be 4.54 × 10^−^^2^ mm^2^. In order to study its response as a function of bias and light intensity, the *I*–*V* curves were measured in dark and under 460 nm light illumination with intensity ranging from 8 × 10^−^^5^ to 310 mW cm^−^^2^ (Fig. 6c). The dark current is measured as low as 1.62 × 10^−^^3^ nA or 3.57 × 10^−^^6^ mA cm^−^^2^ at 2 V bias; the light current of the same device increases to 1068 nA or 2.35 mA cm^−^^2^ under illumination intensity of 10 mW cm^−^^2^, exceeding 5 orders of magnitude enhancement comparing to its dark current. It should be noted that, the dark current of the present (PEA)_2_PbI_4_ SCM device is strikingly lower than the control device made of 3D perovskite single-crystal wafer (Supplementary Table 4). In general, the dark current decreases as the material quality increases. In other words, devices with fewer defects are expected to show lower dark current. To compare the optoelectronic performance between the (PEA)_2_PbI_4_ SCM and MCTF, the *I*–*V* curves of their corresponding devices were measured under illumination, as shown in Supplementary Fig. 21. Under the same illumination condition, photocurrent of the (PEA)_2_PbI_4_ MCTF device measured only 0.12 nA or 2.64 × 10^−^^4^ mA cm^−^^2^, about 8900 times smaller.

The key sensor parameters such as *R*, EQE, and D* are calculated and provided in Supplementary Information. Figure 6d shows the photoresponse as a function of the incident light intensity. Both the photocurrent density and *R* show good linear correlation with high correlation coefficients (0.999). It can be seen that the photocurrent of the SCM devices show a linear response from 8 × 10^−^^5^ to 310 mW cm^−^^2^, with an linear dynamic range (LDR) of 124 dB. Figure 6f shows the EQE for the flexible photosensor based on the (PEA)_2_PbI_4_ SCM within the illumination intensity ranging from 8 × 10^−^^5^ to 310 mW cm^−^^2^ at wavelength of 460 nm under a 4 V bias. To obtain the detectivity (D*), we first directly recorded the noise current by a dynamic signal analyzer, a method that has been well established by Tang et al. and Huang et al.^69,70^. As shown in Fig. 6e, the measured average noise current of the (PEA)_2_PbI_4_ SCM photosensor is approximately 1.29 × 10^−^^15^ A Hz^−^^1/2^. It can be seen that the noise current is independent of the frequency, indicating that the noise of the present devices is not dominated by the 1/*f* noise due to the minimized trap density and the absence of grain boundaries in the (PEA)_2_PbI_4_ SCMs^69,70^. The two main noise sources are the shot noise (*i*_n,s_) and the thermal noise (*i*_n,t_), respectively. The shot noise (*i*_n,s_) can be calculated from the dark current using the following equation:

3 $$i_{{\mathrm{n}},{\mathrm{s}}} = \sqrt {2ei_{\mathrm{d}}B}$$

where *i*_d_ is the dark current, *e* the elementary charge, and *B* the electrical bandwidth. Based on dark current of the device at 4 V bias, the shot noise is calculated to be 9.12 × 10^−^^16^ A Hz^−^^1/2^. Besides the shot noise, the thermal noise (*i*_n,t_) is determined by the following equation:

4 $$i_{{\mathrm{n}},{\mathrm{t}}} = \sqrt {\frac{{4k_{\mathrm{B}}TB}}{R}}$$

where *k*_B_ is the Boltzmann constant, *T* the temperature, and *R* the resistance of the (PEA)_2_PbI_4_ SCM photosensor. Based on the dark current curve, the resistance at 4 V is obtained and so that the thermal noise is calculated to be 1.03 × 10^−^^16^ A Hz^−^^1/2^. Undoubtedly, large resistivity of the photosensor resulted in low dark current, small shot noise, and low thermal noise. The total noise (*i*_n,T_) can be calculated according to the expression below:

5 $$i_{{\mathrm{n}},{\mathrm{T}}} = \sqrt {i_{{\mathrm{n}},{\mathrm{s}}}^2 + i_{{\mathrm{n}},{\mathrm{t}}}^2}$$

As a result, the calculated total noise limit 9.18 × 10^−^^16^ A Hz^−^^1/2^ by only considering the shot noise and thermal noise is very close to the measured noise 1.29 × 10^−^^15^ A Hz^−^^1/2^. Based on the measured dark current noise (*i*_n_) and the responsivity (*R*) of the photosensor, the noise equivalent power (NEP) can be calculated by the following equation:

6 $${\mathrm{NEP}} = \frac{{i_{\mathrm{n}}}}{R}$$

Accordingly, the specific detectivity (D*) of the photosensor is deduced by the following equation:

7 $$D^ \ast = \frac{{\sqrt {AB} }}{{{\mathrm{NEP}}}} = \frac{{{{R}}\sqrt {AB} }}{{i_{\mathrm{n}}}}$$

where *A* is the active area of the device and *B* the electrical bandwidth. Finally, D* is calculated and shown in Fig. 6f as a function of the illumination intensity ranging from 8 × 10^−^^5^ to 310 mW cm^−^^2^ at wavelength of 460 nm with a fixed 4 V bias. Because of more charge recombination occurred under higher illumination intensity, all values of *R*, EQE, and D* decrease as the incident light intensity increases. Figure 6g shows its spectral photocurrent response. As expected, the photosensor shows high response in the wavelength region of 350–550 nm, consistent with its optical absorption. *R*, EQE, and D* against various wavelength under a fixed 4 V bias are calculated and shown in Supplementary Fig. 22. Under the lowest measured incident light, power density achieves 8 × 10^−^^5^ mW cm^−^^2^, and the highest *R*, EQE, and D* are calculated to be 98.17 A W^−^^1^, 2.65 × 10^4^% and 1.62 × 10^15^ cm Hz^1/2^ W^−^^1^ (Jones), respectively. Note that the extraordinarily high D* value of the present device (exceeding 10^15^ cm Hz^1/2^ W^−^^1^) is among the record high reported for the perovskite photosensors so far, and it is even higher than that of devices based on single-crystalline silicon^56^, InGaAs^57^, GaAs^58^, CdTe^59^, and GaN^60^, demonstrating much superior quality of the 2D layered (PEA)_2_PbI_4_ single-crystalline perovskite membrane material.

Supplementary Fig. 23a presents the time-dependent on/off cycle test. Clearly, the device is very stable during the test process under multiple repeated on/off cycles with the bias at 0.5, 1, 2, 3, and 4 V. The photocurrent response as a function of bias voltage of the photosensors is provided in Supplementary Fig. 23b. It is found that there is a good linear correlation with high correlation coefficients (approximately 1). Furthermore, we measured the response time of the (PEA)_2_PbI_4_ SCM photosensors. The rise time (*t*_rise_) is generally defined in literature as time needed for the photocurrent to rise from 10% peak value to 90% and inverse for the decay time (*t*_decay_). As shown in Fig. 6h, the photosensor shows the response speed with the photocurrent rise and fall times as 64 and 52 µs, respectively. Figure 6i depicts the plot of the frequency responses of the photosensor, showing a −3 dB cutoff frequency up to 10.72 KHz. Undoubtedly, the fast photocurrent response of these photosensors can be attributed to very low defect density of the SCM. More detailed comparison is provided in Supplementary Table 5.

Apart from the stability and detectivity, the mechanical flexibility is also an important parameter for the photosensors. To assess the flexibility of the present (PEA)_2_PbI_4_ SCM photosensor, the optoelectronic performance of the device has been measured precisely at various bending angles under constant incident light intensity. The corresponding photocurrents are shown in Supplementary Fig. 24a–f. When compared with the original data given in Supplementary Fig. 24a, the responsivities decrease slightly, and the reduction is calculated to be approximately 8%, even when the sensor is bent to 155° of flexing angle (Supplementary Fig. 24f), thus demonstrating the superior flexibility of the photosensor.

## Discussion

In summary, we have developed an effective process to grow high-quality flexible 2D layered (PEA)_2_PbI_4_ SCM. Under optimal temperature ramp rate, the size of these SCMs is >73 × 35 mm^2^, with thickness as thin as 0.6 μm. As far as we know, this is the first time to grow flexible perovskite SCM with thickness thinner than 10 μm and area exceeding 2500 mm^2^. XRD, GIWAXS, and TEM analyses confirm that the SCMs are of layered structure. In addition, the SCM displays very low defect density, long-term stability, and excellent flexibility. It is also found that the as-synthesized (PEA)_2_PbI_4_ SCM shows excitation-dependent fluorescence behavior. Finally, the flexible perovskite SCM with large surface area opens a way for its application in high-performance flexible planar photosensors. The successful growth and further utilization of the flexible 2D layered (PEA)_2_PbI_4_ SCM not only provide a new material system but also open up an avenue to applications in optoelectronics.

## Methods

### Materials

GBL (99%) and Lead iodide (PbI_2_, 99%) were purchased from Aladdin Reagent Ltd. Phenylethylammonium iodide (PEAI, 99%) was purchased from Xi’an Polymer Light Technology Corp. All the chemicals were used as received without further purification.

### Fabrication of the (PEA)_2_PbI_4_ single-crystal membranes (SCMs)

Induced peripheral crystallization procedure is schematically illustrated in Fig. 2a. Briefly, PbI_2_ and PEAI (1:2 molar ratio) were dissolved in GBL at 80 °C under active mixing for 24 h to generate the (PEA)_2_PbI_4_ precursor solution (2.12 M). The solution was then filtered prior to crystal growth. For the growth of the (PEA)_2_PbI_4_ SCM, a drop of the prepared precursor solution was pipetted onto a glass slide substrate (or flexible plastic substrate, PET substrate) preheated to 80 °C as shown in step 1. Then a second glass slide with smaller lateral dimensions was placed onto the precursor solution drop. The solution was squeezed to spread evenly between two glass slides (step 2). It should be noted that part of the precursor solution was squeezed out to the edge of the top slide. Step 3 is to maintain the temperature of the entire assembly at 80 °C in an enclosed oven. In such case, the evaporation the precursor solution along the edge of the top slide was well controlled at a consistent rate. In about 2h, nucleation and crystallization occurred at the edge of the top slide, as evidenced by some shinny crystallites formed along the edges of the second slide. Further gradient decrease of the temperature from 80 to 30 °C at a rate of 1 °C h^−1^ led to the growth of the (PEA)_2_PbI_4_ SCM between the slides (step 4).

### Fabrication of the (PEA)_2_PbI_4_ MCTFs

The glass substrate was cleaned by successive sonication with acetone, isopropanol, and ethanol for 30 min and then dried with N_2_ flow. The clean substrate was then treated with UV-Ozone for 15 min before solution casting. Precursor solution (70 μL) was dropped on the fresh-prepared glass substrate followed by spin-coating at 5000 r.p.m. for 20 s without delay. The as-cast films were then annealed at 100 °C for 10 min. The spin-coating was accomplished under inert atmosphere inside a nitrogen-filled glove box.

### Fabrication of devices for relative dielectric constant (*ε*) measurements

Seven devices were fabricated by depositing Au electrodes (100-nm thickness) on two opposite surfaces of the (PEA)_2_PbI_4_ SCMs.

### Fabrication of devices for SCLC measurements

The devices were fabricated by depositing the Au electrodes (approximately 100-nm thickness) with the architecture of Au/(PEA)_2_PbI_4_ SCM/Au for hole-only devices and Au/PCBM/(PEA)_2_PbI_4_ SCM/PCBM/Au for electron-only devices.

### Fabrication of the (PEA)_2_PbI_4_ SCM planar photodetectors

Planar photodetectors were fabricated by depositing interdigital Au electrodes (200-nm thickness) via vacuum evaporation method on the (PEA)_2_PbI_4_ SCMs and (PEA)_2_PbI_4_ MCTFs. The bridging gap of electrode width is 20 μm, while the effective illuminated area of each device was about 4.54 × 10^−^^2^ mm^2^.

### Characterization

Powder XRD and high-resolution XRD on SC wafer: XRD patterns were collected using a Bruker D8 Advance X-ray diffractometer equipped with a Cu tube (*λ* = 1.5406 Å) operated at 40 kV and 20 mA. High-resolution XRD measurement was taken using X’Pert MRD, with Cu Kα1 line (*λ* = 1.5406 Å) with *V* = 40 KV and *I* = 20 mA.

Grazing-incidence wide angle X-ray scattering: GIWAXS measurements were performed at D line at the Cornell High Energy Synchrotron Source. The wavelength of the X-rays was 1.157 Å with a bandwidth Δ*λ/λ* of 1.5%. The scattering signal was collected by Pilatus 200 K detector, with a pixel size of 172 × 172 µm^2^ placed at 191 mm away from the sample position. The incident angle of the X-ray beam was at 0.30° and the integration time was 1 s.

Thermal analysis: TGA was performed on a TA SDT-Q600 V20.9 (Build 20). The sample was placed in an Al_2_O_3_ crucible and heated in an interval from room temperature to 800 °C at a ramp rate 5 °C min^−1^ under flowing nitrogen gas with a flow rate 100 mL min^−1^. About 3 mg of (PEA)_2_PbI_4_ SCM powder was used for the TGA measurements.

UV-Vis-near infrared (NIR) absorbance spectra measurements: UV-Vis absorbance spectrum of (PEA)_2_PbI_4_ SCMs and (PEA)_2_PbI_4_ MCTFs were measured using a Perkin-Elmer Lambda 950 UV-Vis-NIR spectrophotometer equipped with an integrating sphere attachment operating in the 300–800 nm region at room temperature.

UPS measurements: UPS was performed on a photoelectron spectrometer (ESCALAB 250Xi, Thermo Fisher Scientific) to study the working function of the (PEA)_2_PbI_4_ SCMs and (PEA)_2_PbI_4_ MCTFs.

Steady-state and time-resolved PL measurements: Steady-state and time-resolved PL measurements of (PEA)_2_PbI_4_ SCMs and (PEA)_2_PbI_4_ MCTFs were taken using a PicoQuant FT-300 and FT-100, with 375 nm excitation wavelength. All PL spectra were measured in air without encapsulation.

Relative dielectric constant measurements: Capacitance of the (PEA)_2_PbI_4_ SCMs were determined using the impedance analyzer (Agilent 4294 A Precision LCR Meter) over a wide frequency range from 100 kHz up to 30 MHz (Figure S10a). The relative dielectric constant of the prepared samples was calculated using the capacitance.

SCLC measurements: The single carrier devices were fabricated and used to measure the trap-state density by using the SCLC method for the (PEA)_2_PbI_4_ SCMs. The structure is Au/(PEA)_2_PbI_4_ SCM/Au for hole-only devices and Au/PCBM/(PEA)_2_PbI_4_ SCM/PCBM/Au for electron-only devices. The dark *I*–*V* curve was measured using the Keithley 4200 semiconductor characterization system and was used to calculate the trap-state density.

Photosensor performance measurements: All device performance characterizations were done in a dark box with optical and electrical shielding to minimize electromagnetic and ambient light disturbance. The photoresponse characteristics were measured using the Keithley 4200 semiconductor characterization system and a manual probe station under various bias voltages. To measure the response speed, a 462 nm semiconductor laser driven by a signal generator (Tektronix, AFG3252C) was used as the light source to generate pulsed laser beam, and the temporal response of the device was measured by using a Low-Noise Current Preamplifier (Stanford Research System, SR570) with a Mixed Domain Oscilloscope (Tektronix, MDO3104). For wavelength-dependent photocurrent measurement, the spectrum was generated by modulating a xenon lamp equipped with a monochromator. Noise currents were measured by using a spectrum analyzer (Keysight 35670 A). A resistor (10 MΩ) was connected in series with the device and the frequency analyzer was connected in parallel with the device. The signal can be read out as V Hz^−^^1/2^ from the frequency analyzer in the range of ~10–1000 Hz, and the corresponding current was calculated by dividing the resistance of the perovskite SCM. The incident intensity was measured using an Optical Power Meter (VEGA OPHIR PD300-UV) and calibrated with a silicon photodetector. All measurements were taken at room temperature.

Electron microscopy: SEM imaging was performed on a field-emission SEM (SU-8020, Hitachi). TEM and HRTEM imaging was conducted on a FEI Tecnai G2 F20.

Atomic force microscopy (AFM): AFM images were acquired on a Bruker Dimension ICON instrument.

Photography: Photographs in this manuscript were taken by using an eight-mega-pixel digital camera.

## Electronic supplementary material

Supplementary Information

## Data Availability

The data that support the plots within this Article and other findings of this study are available from the corresponding authors upon reasonable request.

## References

[1] Liu Y (2016). Thinness- and shape-controlled growth for ultrathin single-crystalline perovskite wafers for mass production of superior photoelectronic devices. Adv. Mater..

[2] Liu Y (2016). 20-mm-Large single-crystalline formamidinium-perovskite wafer for mass production of integrated photodetectors. Adv. Opt. Mater..

[3] Liu Y (2015). Two-inch-sized perovskite CH_3_NH_3_PbX_3_ (X = Cl, Br, I) crystals: growth and characterization. Adv. Mater..

[4] Zhang F (2017). Perovskite CH_3_NH_3_PbI_3-x_Br_x_ single crystals with charge-carrier lifetimes exceeding 260 μs. ACS Appl. Mater. Interfaces.

[5] Shi D (2015). Low trap-state density and long carrier diffusion in organolead trihalide perovskite single crystals. Science.

[6] Saidaminov MI (2015). High-quality bulk hybrid perovskite single crystals within minutes by inverse temperature crystallization. Nat. Commun..

[7] Dong Q (2015). Electron-hole diffusion lengths >175 mum in solution-grown CH3NH3PbI3 single crystals. Science.

[8] Zhang F, Yang B, Li Y, Deng W, He R (2017). Extra long electron–hole diffusion lengths in CH_3_NH_3_PbI_3−x_Cl_x_ perovskite single crystals. J. Mater. Chem. C.

[9] Liu Y, Yang Z, Liu SZ (2018). Recent progress in single-crystalline perovskite research including crystal preparation, property evaluation, and applications. Adv. Sci..

[10] Nayak PK (2016). Mechanism for rapid growth of organic-inorganic halide perovskite crystals. Nat. Commun..

[11] Uribe JI, Ramirez D, Osorio-Guillén JM, Osorio J, Jaramillo F (2016). CH_3_NH_3_CaI_3_ perovskite: synthesis, characterization, and first-principles studies. J. Phys. Chem. C.

[12] Dang Y (2016). Formation of hybrid perovskite tin iodide single crystals by top-seeded solution growth. Angew. Chem. Int. Ed..

[13] Yao Z (2017). Local temperature reduction induced crystallization of MASnI_3_ and achieving a direct wafer production. RSC Adv..

[14] Zhumekenov AA (2016). Formamidinium lead halide perovskite crystals with unprecedented long carrier dynamics and diffusion length. Acs Energy Lett..

[15] Han Q (2016). Single crystal formamidinium lead iodide (FAPbI_3_): insight into the structural, optical, and electrical properties. Adv. Mater..

[16] Saidaminov MI, Abdelhady AL, Maculan G, Bakr OM (2015). Retrograde solubility of formamidinium and methylammonium lead halide perovskites enabling rapid single crystal growth. Chem. Commun..

[17] Dang Y (2016). Crystallographic investigations into properties of acentric hybrid perovskite single crystals NH(CH_3_)_3_SnX_3_ (X = Cl, Br). Chem. Mater..

[18] Sakai N (2017). Solution-processed cesium hexabromopalladate(IV), Cs_2_PdBr_6_, for optoelectronic applications. J. Am. Chem. Soc..

[19] Slavney AH, Hu T, Lindenberg AM, Karunadasa HI (2016). A bismuth-halide double perovskite with long carrier recombination lifetime for photovoltaic applications. J. Am. Chem. Soc..

[20] McClure ET, Ball MR, Windl W, Woodward PM (2016). Cs_2_AgBiX_6_(X = Br, Cl): new visible light absorbing, lead-free halide perovskite semiconductors. Chem. Mater..

[21] Dirin DN, Cherniukh I, Yakunin S, Shynkarenko Y, Kovalenko MV (2016). Solution-grown CsPbBr_3_ perovskite single crystals for photon detection. Chem. Mater..

[22] Song J (2017). Ultralarge all-inorganic perovskite bulk single crystal for high-performance visible-infrared dual-modal photodetectors. Adv. Opt. Mater..

[23] Stoumpos CC (2013). Crystal growth of the perovskite semiconductor CsPbBr_3_: a new material for high-energy radiation detection. Cryst. Growth Des..

[24] Saidaminov MI (2017). Inorganic lead halide perovskite single crystals: phase-selective low-temperature growth, carrier transport properties, and self-powered photodetection. Adv. Opt. Mater..

[25] Zhang Y, Liu Y, Li Y, Yang Z, Liu SZ (2016). Perovskite CH_3_NH_3_Pb(Br_x_I_1−x_)_3_ single crystals with controlled composition for fine-tuned bandgap towards optimized optoelectronic applications. J. Mater. Chem. C.

[26] Wei H (2017). Dopant compensation in alloyed CH_3_NH_3_PbBr_3-x_Cl_x_ perovskite single crystals for gamma-ray spectroscopy. Nat. Mater..

[27] Miao X (2017). Air-stable CsPb_1−x_Bi_x_Br_3_ (0 ≤ x≪1) perovskite crystals: optoelectronic and photostriction properties. J. Mater. Chem. C.

[28] Huang Y (2017). The intrinsic properties of FA_(1−x)_MA_x_PbI_3_ perovskite single crystals. J. Mater. Chem. A.

[29] Zhang T (2015). A facile solvothermal growth of single crystal mixed halide perovskite CH_3_NH_3_Pb(Br_1-x_Cl_x_)_3_. Chem. Commun..

[30] Liu Y (2017). 120 mm Single-crystalline perovskite and wafers: towards viable applications. Sci. China Chem..

[31] Cao F (2018). Novel perovskite/TiO_2_/Si trilayer heterojunctions for high-performance self-powered ultraviolet-visible-near infrared (UV-Vis-NIR) photodetectors. Nano Res..

[32] Sun H (2017). Self-powered, flexible, and solution-processable perovskite photodetector based on low-cost carbon cloth. Small.

[33] Lu H (2016). A self-powered and stable all-perovskite photodetector-solar cell nanosystem. Adv. Funct. Mater..

[34] Peng W (2016). Solution-grown monocrystalline hybrid perovskite films for hole-transporter-free solar cells. Adv. Mater..

[35] Rao HS, Chen BX, Wang XD, Kuang DB, Su CY (2017). A micron-scale laminar MAPbBr_3_ single crystal for an efficient and stable perovskite solar cell. Chem. Commun..

[36] Bao C (2017). Low-noise and large-linear-dynamic-range photodetectors based on hybrid-perovskite thin-single-crystals. Adv. Mater..

[37] Chen Z (2017). Thin single crystal perovskite solar cells to harvest below-bandgap light absorption. Nat. Commun..

[38] Rao HS, Li WG, Chen BX, Kuang DB, Su CY (2017). In situ growth of 120 cm^2^ CH_3_NH_3_PbBr_3_ perovskite crystal film on FTO glass for narrowband-photodetectors. Adv. Mater..

[39] Zhang X (2017). Stable high efficiency two-dimensional perovskite solar cells via cesium doping. Energy Environ. Sci..

[40] Thirumal K (2017). Morphology-independent stable white-light emission from self-assembled two-dimensional perovskites driven by strong exciton–phonon coupling to the organic framework. Chem. Mater..

[41] Ha ST, Shen C, Zhang J, Xiong Q (2015). Laser cooling of organic-inorganic lead halide perovskites. Nat. Photon..

[42] Gauthron, K. et al. Optical spectroscopy of two-dimensional layered (C_6_H_5_C_2_H_4_NH_3_)_2_PbI_4_ perovskite. *Opt. Express* **18**, 5912–5919 (2010). 10.1364/OE.18.005912 20389609

[43] Mitzi DB (1999). A layered solution crystal growth technique and the crystal structure of (C_6_H_5_C_2_H_4_NH_3_)_2_PbCl_4_. J. Solid State Chem..

[44] Kagan CR (1999). Organic-inorganic hybrid materials as semiconducting channels in thin-film field-effect transistors. Science.

[45] Liang K, Mitzi DB, Prikas MT (1998). Synthesis and characterization of organic−inorganic perovskite thin films prepared using a versatile two-step dipping technique. Chem. Mater..

[46] Era M, Morimoto S, Tsutsui T, Saito S (1994). Organic‐inorganic heterostructure electroluminescent device using a layered perovskite semiconductor (C_6_H_5_C_2_H_4_NH_3_)_2_PbI_4_. Appl. Phys. Lett..

[47] Dammak T (2009). Two-dimensional excitons and photoluminescence properties of the organic/inorganic (4-FC_6_H_4_C_2_H_4_NH_3_)_2_[PbI_4_] nanomaterial. J. Phys. Chem. C.

[48] Kitazawa N (1997). Optical absorption and photoluminescence properties of Pb(I, Br)-based two-dimensional layered perovskite. Jpn. J. Appl. Phys..

[49] Kitazawa N (1997). Excitons in two-dimensional layered perovskite compounds: (C_6_H_5_C_2_NH_3_)_2_H_4_ Pb(Br,I)_4_ and (C_6_H_5_C_2_NH_3_Pb(Cl,Br)_4_. Mater. Sci. Eng. B.

[50] Kikuchi K, Takeoka Y, Rikukawa M, Sanui K (2004). Structure and optical properties of lead iodide based two-dimensional perovskite compounds containing fluorophenethylamines. Curr. Appl. Phys..

[51] Saidaminov MI, Mohammed OF, Bakr OM (2017). Low-dimensional-networked metal halide perovskites: the next big thing. ACS Energy Lett..

[52] Shimizu M, Fujisawa Ji, Ishihara T (2006). Photoluminescence of the inorganic-organic layered semiconductor(C_6_H_5_C_2_H_4_NH_3_)_2_PbI_4_: observation of triexciton formation. Phys. Rev. B.

[53] Peng W (2017). Ultralow self-doping in two-dimensional hybrid perovskite single crystals. Nano. Lett..

[54] Liu W (2017). Giant two-photon absorption and its saturation in 2D organic-inorganic perovskite. Adv. Opt. Mater..

[55] Lédée F (2017). Fast growth of monocrystalline thin films of 2D layered hybrid perovskite. CrystEngComm.

[56] Dou L (2014). Solution-processed hybrid perovskite photodetectors with high detectivity. Nat. Commun..

[57] Gong X (2009). High-detectivity polymer photodetectors with spectral response from 300 nm to 1450 nm. Science.

[58] Ghadi H (2015). Enhancement in peak detectivity and operating temperature of strain-coupled InAs/GaAs quantum dot infrared photodetectors by rapid thermal annealing. IEEE Trans. Nanotechnol..

[59] Wei H, Fang Y, Yuan Y, Shen L, Huang J (2015). Trap engineering of CdTe nanoparticle for high gain, fast response, and low noise P3HT:CdTe nanocomposite photodetectors. Adv. Mater..

[60] Gundimeda A (2017). Fabrication of non-polar GaN based highly responsive and fast UV photodetector. Appl. Phys. Lett..

[61] Dang YY (2015). Bulk crystal growth of hybrid perovskite material CH_3_NH_3_PbI_3_. CrystEngComm.

[62] Nernst W (1904). Theorie der reaktionsgeschwindigkeit in heterogenen systemen. Z. Phys. Chem..

[63] Dash JG (1977). Clustering and percolation transitions in helium and other thin films. Phys. Rev. B.

[64] Sung MH, Kim JS, Kim WS, Hirasawa I, Kim WS (2002). Modification of crystal growth mechanism of yttrium oxalate in metastable solution. J. Cryst. Growth.

[65] Zhang NH, Xing JJ (2006). An alternative model for elastic bending deformation of multilayered beams. J. Appl. Phys..

[66] Li JB (2017). Solution coating of superior large-area flexible perovskite thin films with controlled crystal packing. Adv. Opt. Mater..

[67] Edri E (2014). Elucidating the charge carrier separation mechanism in CH_3_NH_3_PbI_3-x_Cl_x_ perovskite solar cells. Nat. Commun..

[68] Konstantatos G, Clifford J, Levina L, Sargent EH (2007). Sensitive solution-processed visible-wavelength photodetectors. Nat. Photon..

[69] Pan W (2017). Cs_2_AgBiBr_6_ single-crystal X-ray detectors with a low detection limit. Nat. Photon..

[70] Fang YJ, Dong QF, Shao YC, Yuan YB, Huang JS (2015). Highly narrowband perovskite single-crystal photodetectors enabled by surface-charge recombination. Nat. Photon..

