# Supplementary material for: Multi-inch single-crystalline perovskite membrane for high-detectivity flexible photosensors
Source: Nat Commun. 2018 Dec 13;9:5302. doi: 10.1038/s41467-018-07440-2 (PMC6294256; doi:10.1038/s41467-018-07440-2)
Supplement: Supplementary file 1 — Supplementary Information [file 41467_2018_7440_MOESM1_ESM.pdf]

Supplementary Information

Multi-Inch Single-Crystalline Perovskite Membrane for High-Detectivity Flexible Photosensors

Yucheng Liu[1]#, Yunxia Zhang[1]#, Zhou Yang[1], Haochen Ye[1], Jiangshan Feng[1], Zhuo Xu[1], Xu Zhang[2], Rahim Munir[3], Jia Liu[1], Ping Zuo[1], Qingxian Li[1], Mingxin Hu[1], Lina Meng[1], Kang Wang[1], Detlef-M. Smilgies[4], Guangtao Zhao[1], Hua Xu[1], Zupei Yang[1], Aram Amassian[3], Jiawei Li[5], Kui Zhao[1]★ & Shengzhong(Frank) Liu[1,2]★

[1]*Key Laboratory of Applied Surface and Colloid Chemistry, Ministry of Education; Shaanxi Key Laboratory for Advanced Energy Devices; Shaanxi Engineering Lab for Advanced Energy Technology; Institute for Advanced Energy Materials; School of Materials Science and Engineering, Shaanxi Normal University, Xi'an 710119, P. R. China.*

[2]*iChEM, Dalian National Laboratory for Clean Energy; Dalian Institute of Chemical Physics, Chinese Academy of Sciences, Dalian, 116023, P. R. China.*

[3]*Division of Physical Sciences and Engineering, Solar and Photovoltaics Engineering Center, King Abdullah University of Science and Technology (KAUST), Thuwal 23955-6900, Kingdom of Saudi Arabia.*

[4]*Cornell High Energy Synchrotron Source, Cornell University, Ithaca, NY 14850, USA.*

[5]*School of Physics and Information Technology, Shaanxi Normal University, Xi'an 710119, P. R. China.*

Correspondence and request for materials should be addressed to S. L. (szliu@dicp.ac.cn) or K. Z. (zhaok@snnu.edu.cn)

Supplementary Figures

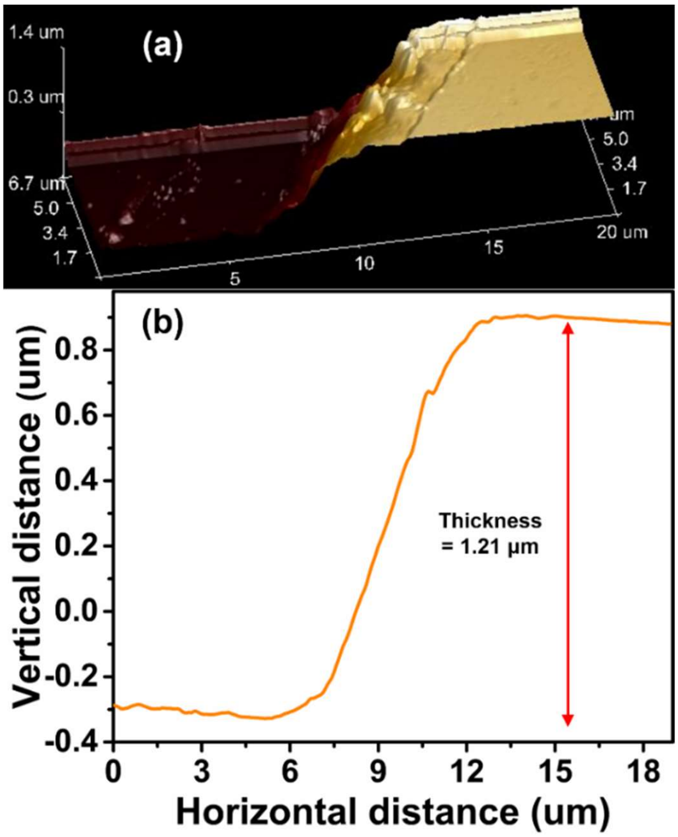

Supplementary Figure 1. (**a**) 3D AFM topographic of a $(PEA)_2PbI_4$ SCM and (**b**) height profile of the line scan.

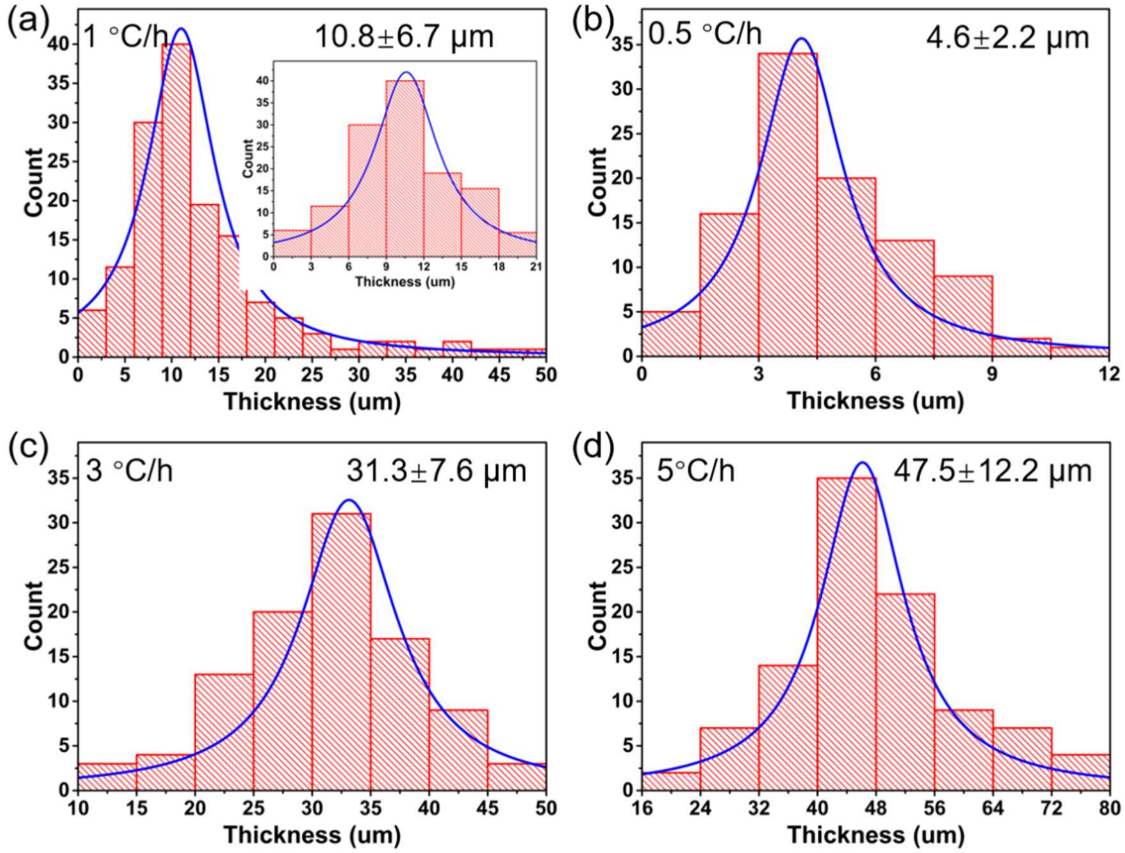

Supplementary Figure 2. Thickness distribution of the $(PEA)_2PbI_4$ SCMs obtained with the cooling rates of (**a**) 1 °C h^{-1}, (**b**) 0.5 °C h^{-1}, (**c**)3 °C h^{-1} and (**d**) 5 °C h^{-1}.

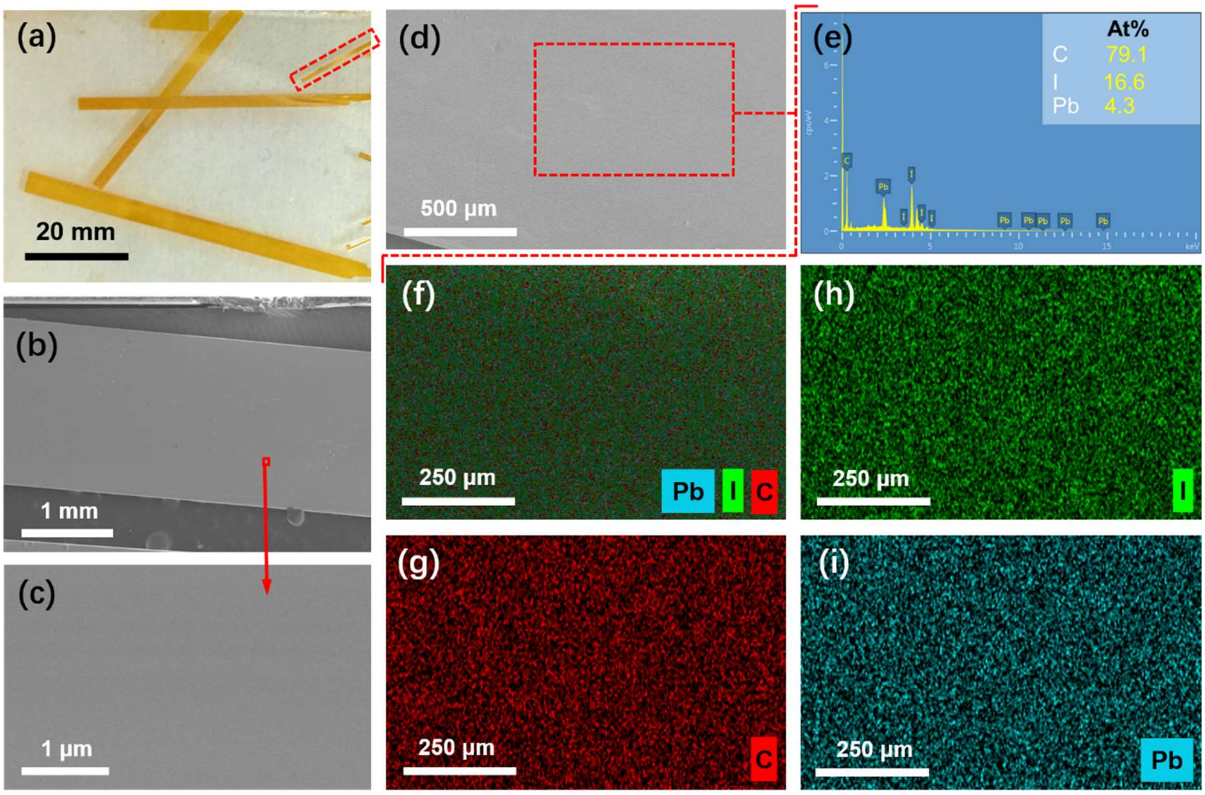

Supplementary Figure 3. (**a**) The photo and (**b**) SEM image of $(PEA)_2PbI_4$ SCMs. (**c**) Higher resolution SEM image of a $(PEA)_2PbI_4$ SCM. (**d, e**) SEM image and EDS spectrum of the three detected elements C, Pb, and I. (**f-i**) EDS mapping scanning measurement of the three detected elements C, Pb, and I.

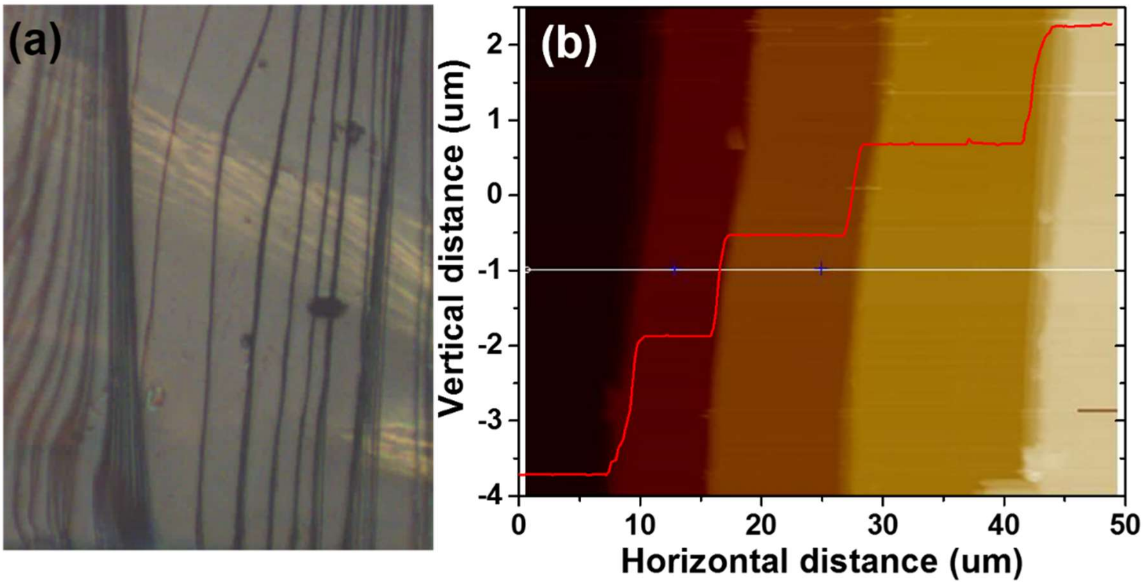

Supplementary Figure 4. (**a**) Optical image and AFM height image of a representative $(PEA)_2PbI_4$ SCM, showing some terraces on the edge. The inset of (**b**) shows the height profile of the image.

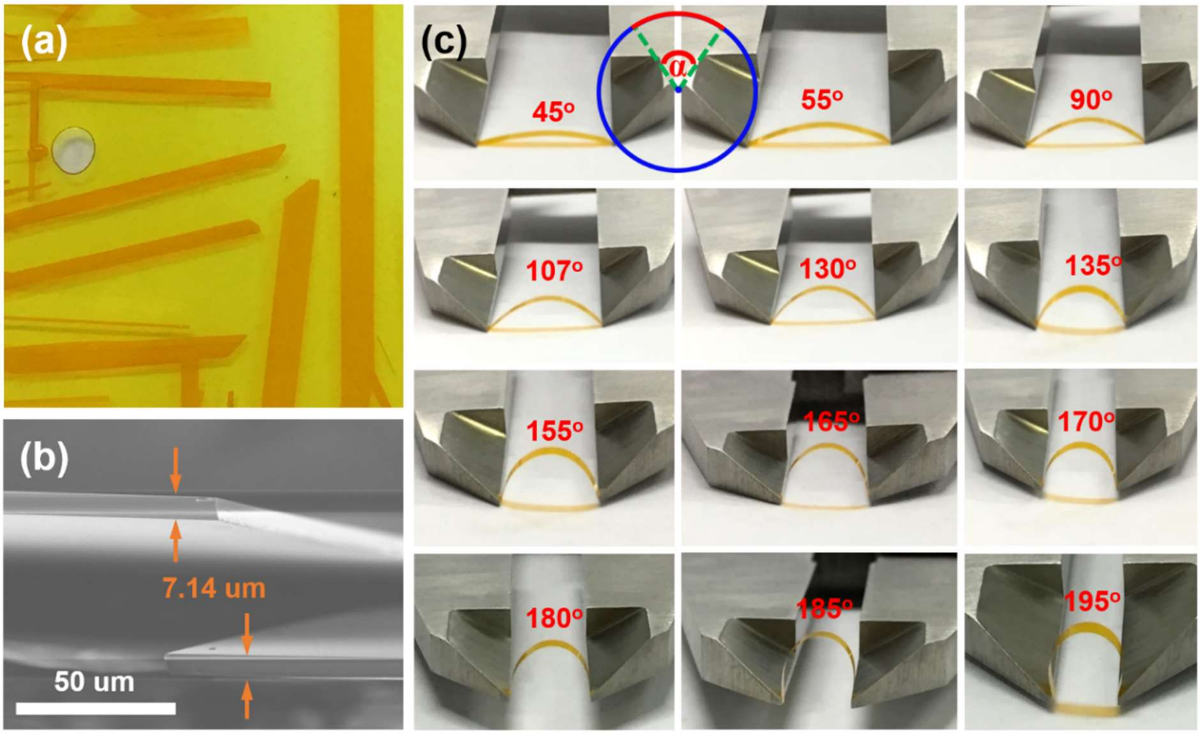

Supplementary Figure 5. (**a**) Photo of the $(PEA)_2PbI_4$ SCMs used for flexing angle measurement. (**b**) Cross-sectional SEM images of a $(PEA)_2PbI_4$ SCM with thicknesses ~7.14 μm. (**c**) The photograph of a $(PEA)_2PbI_4$ SCM bent at flexing angles from 45° to 195°.

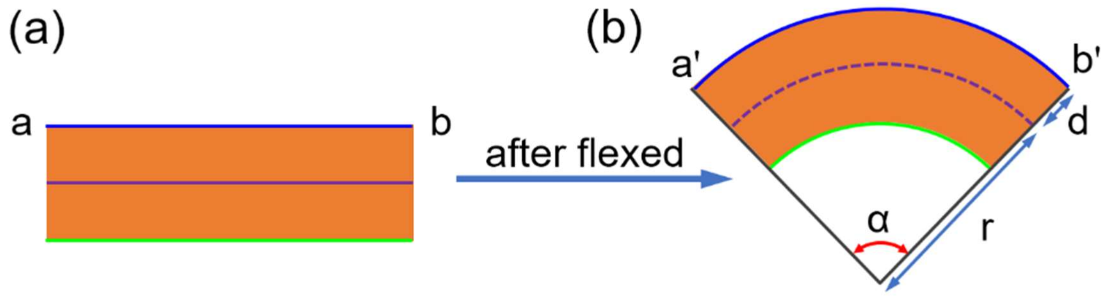

Supplementary Figure 6. Schematic illustration of the $(PEA)_2PbI_4$ SCM (**a**) before and (**a**) after flexed.

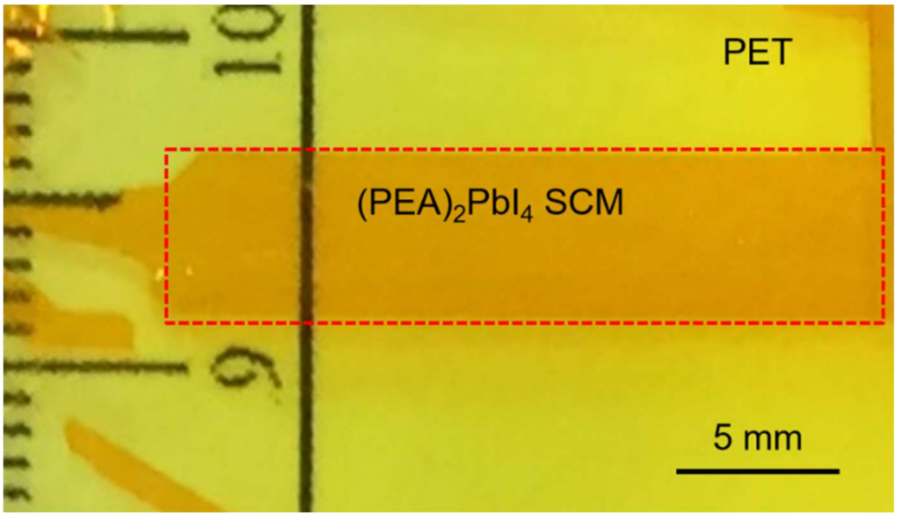

Supplementary Figure 7. A photo of a $(PEA)_2PbI_4$ SCM on flexible PET substrate.

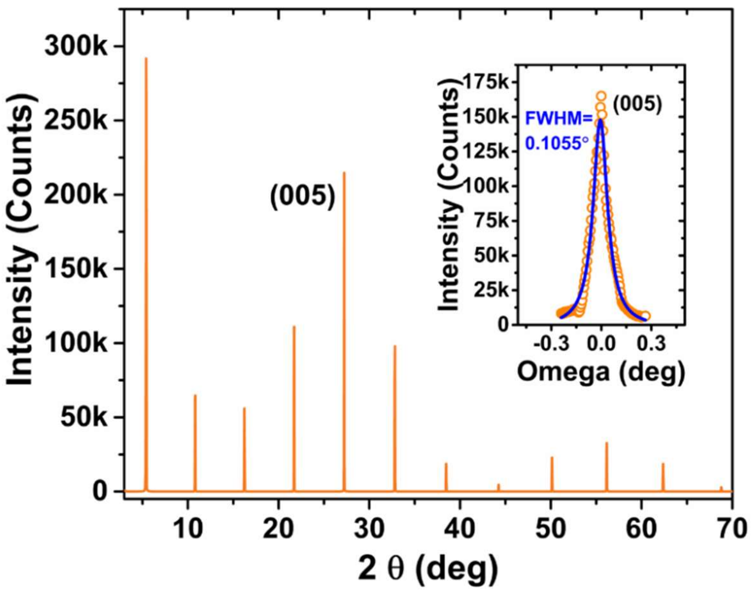

Supplementary Figure 8. High-resolution X-ray diffraction of a $(PEA)_2PbI_4$ SCM with the inset showing the rocking-curve corresponding to the (005) plane.

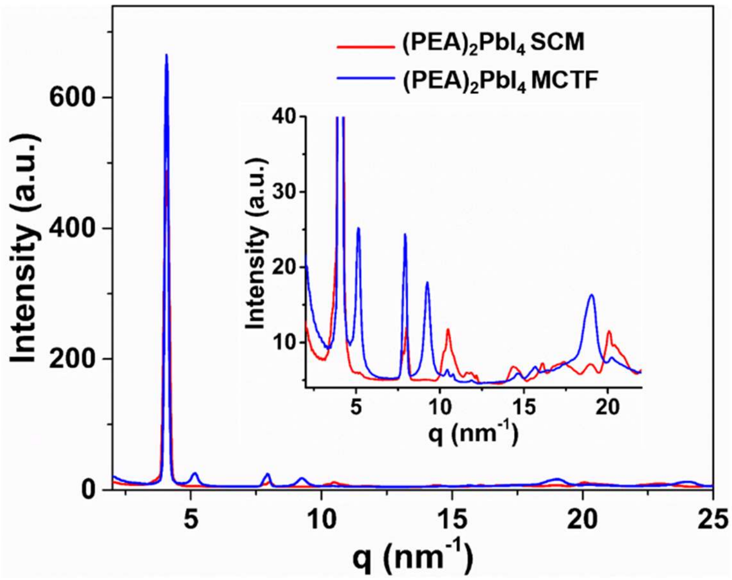

Supplementary Figure 9. Integrated GIWAXS spectra of the $(PEA)_2PbI_4$ SCM and $(PEA)_2PbI_4$ MCTF.

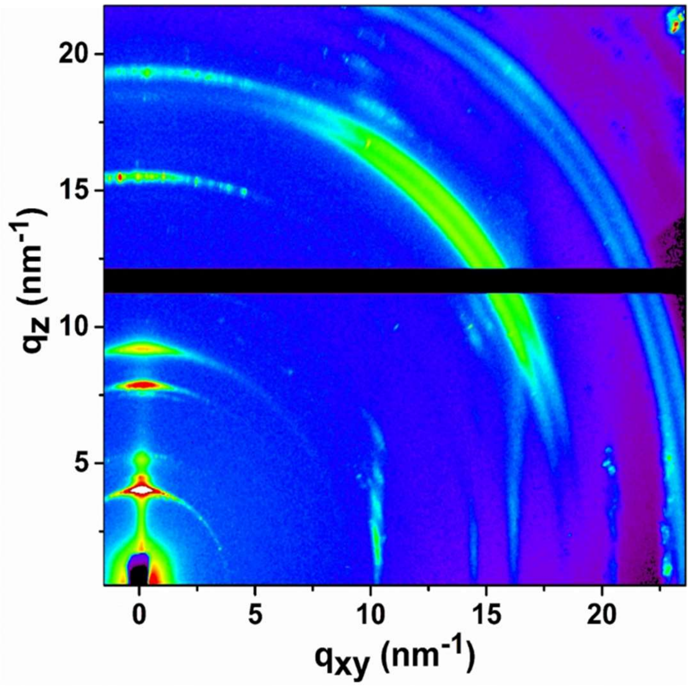

Supplementary Figure 10. GIWAXS image of the $(PEA)_2PbI_4$ MCTF.

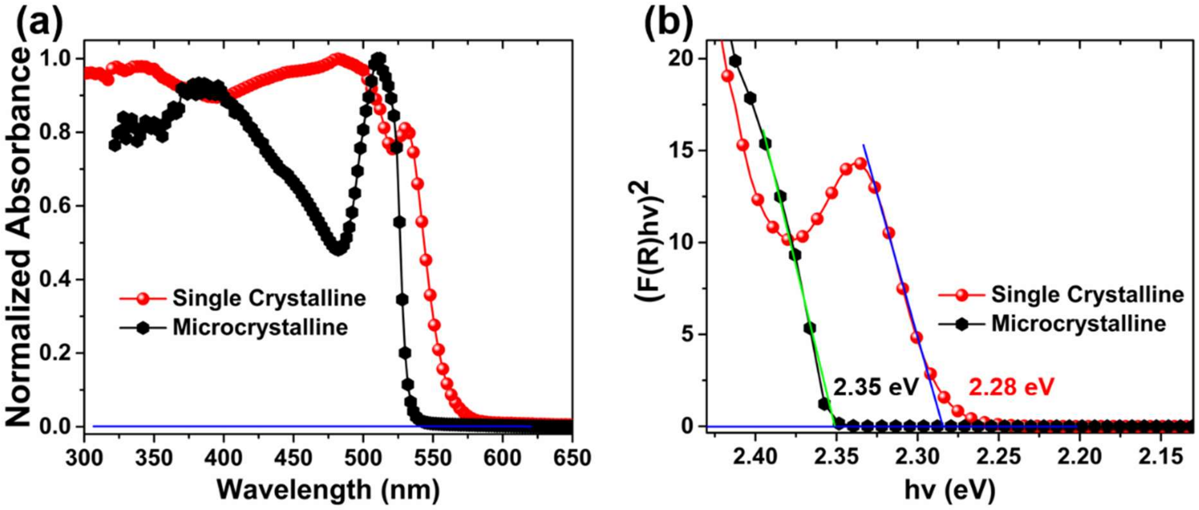

Supplementary Figure 11. The absorption spectra (**a**) and corresponding Tauc plot (**b**) of the $(PEA)_2PbI_4$ SCM and $(PEA)_2PbI_4$ MCTF.

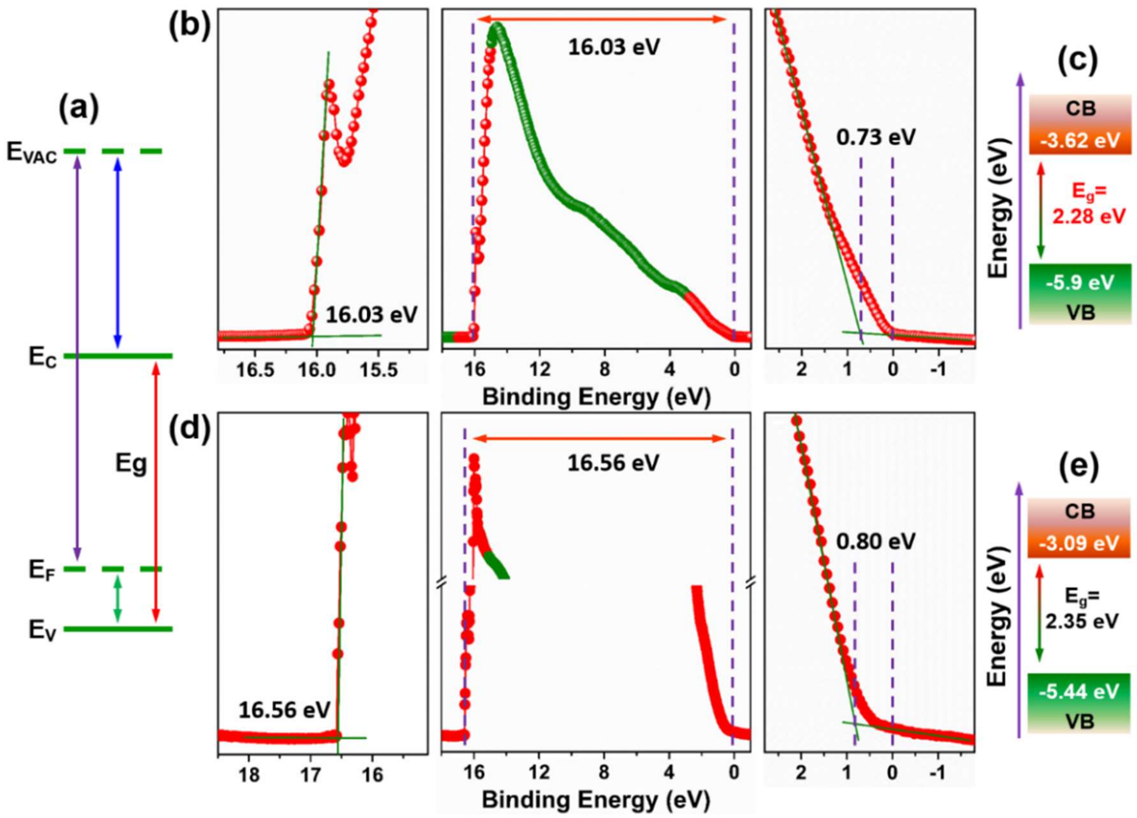

Supplementary Figure 12. (**a**) Schematic illustration of the energy diagram. (**b, d**) The Ultraviolet photoemission spectroscopy spectrum of the $(PEA)_2PbI_4$ SCM and $(PEA)_2PbI_4$ MCTF. The left panel shows the magnified view of the high binding energy region and the right panel shows the magnified view of the low binding energy region. (**c, e**) Energy band diagram for the $(PEA)_2PbI_4$ SCM and $(PEA)_2PbI_4$ MCTF drawn according to UPS results.

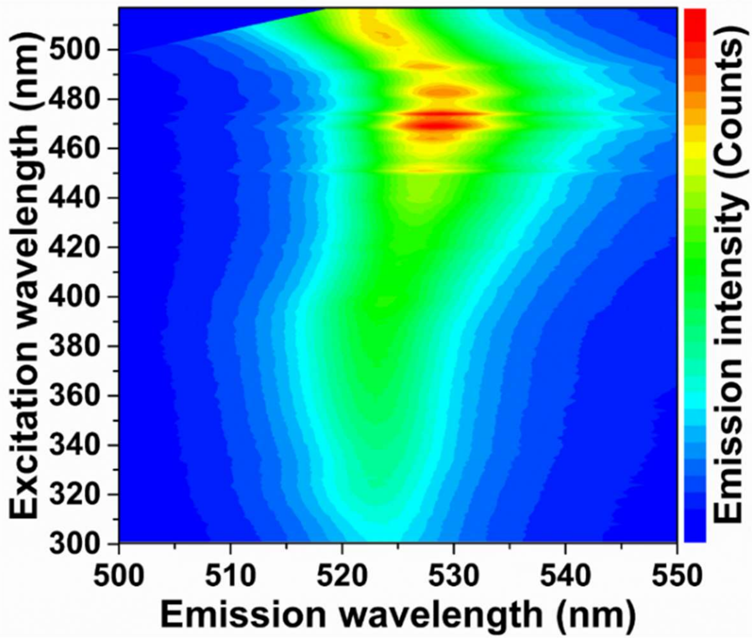

Supplementary Figure 13. 3D excitation-emission plot for the $(PEA)_2PbI_4$ MCTF. Emission intensity rises with the color changing from blue to green and to red.

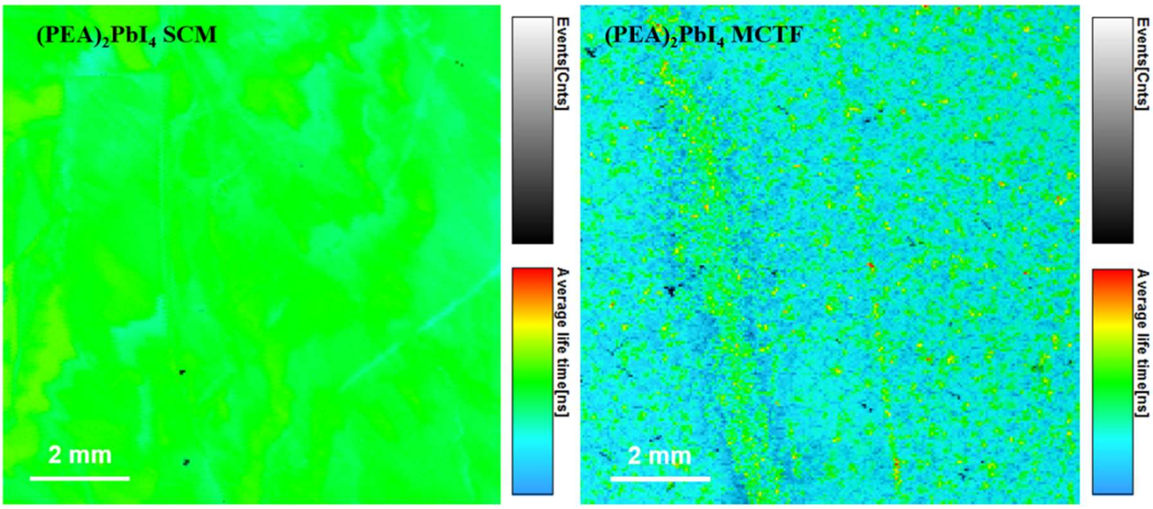

Supplementary Figure 14. PL mapping of the $(PEA)_2PbI_4$ SCM (**a**) and $(PEA)_2PbI_4$ MCTF (**b**) excited at 375 nm.

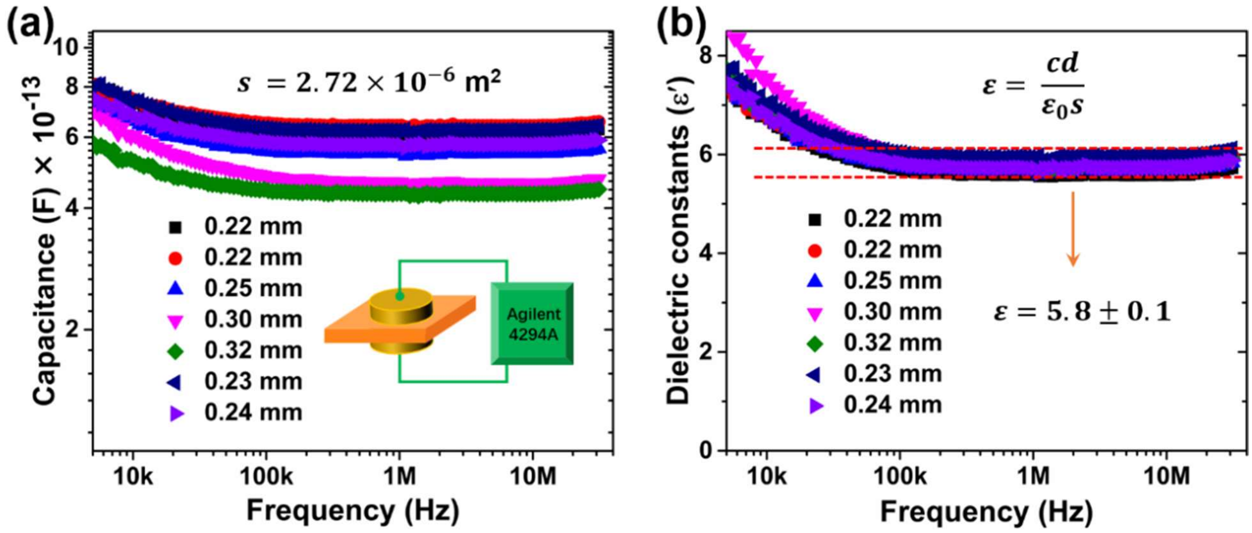

Supplementary Figure 15. (**a**) The capacitance and (**b**) dielectric constant dependent frequency curves of the $(PEA)_2PbI_4$ SCMs. Seven devices were fabricated by depositing ~100 nm thick Au on two opposite surfaces of the $(PEA)_2PbI_4$ SCMs and measured under same condition. The thickness of the SCMs are 0.22 mm, 0.22 mm, 0.25 mm, 0.30 mm, 0.32 mm, 0.23 mm and 0.24 mm, respectively. The area is 2.72×10^{-6} m^2.

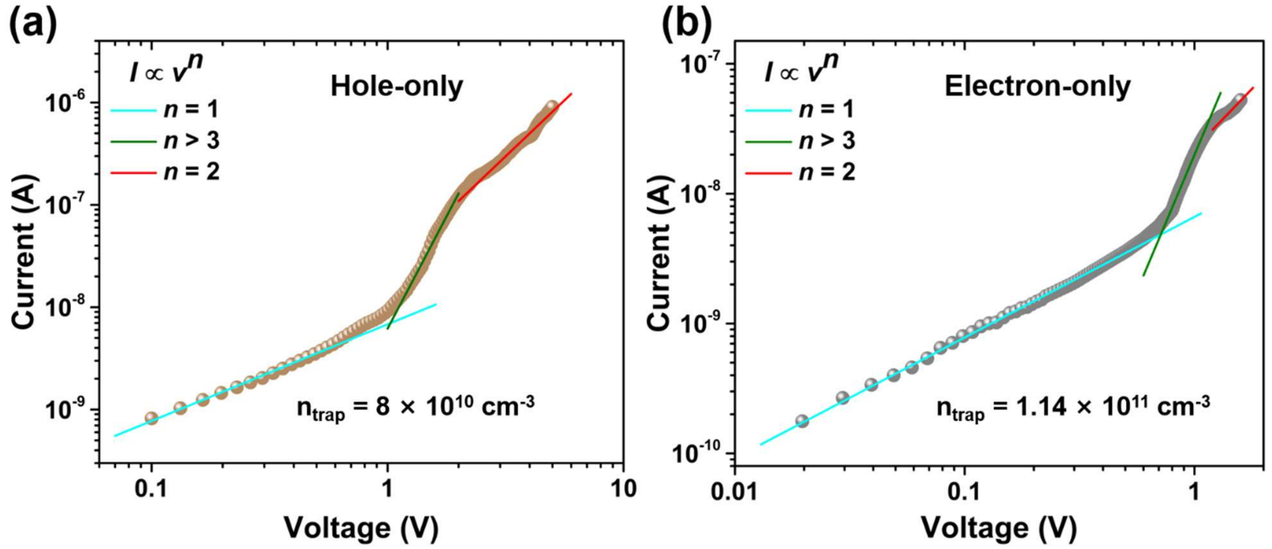

Supplementary Figure 16. Current-voltage curves of the (**a**) hole-only and (**b**) electron-only $(PEA)_2PbI_4$ SCM devices.

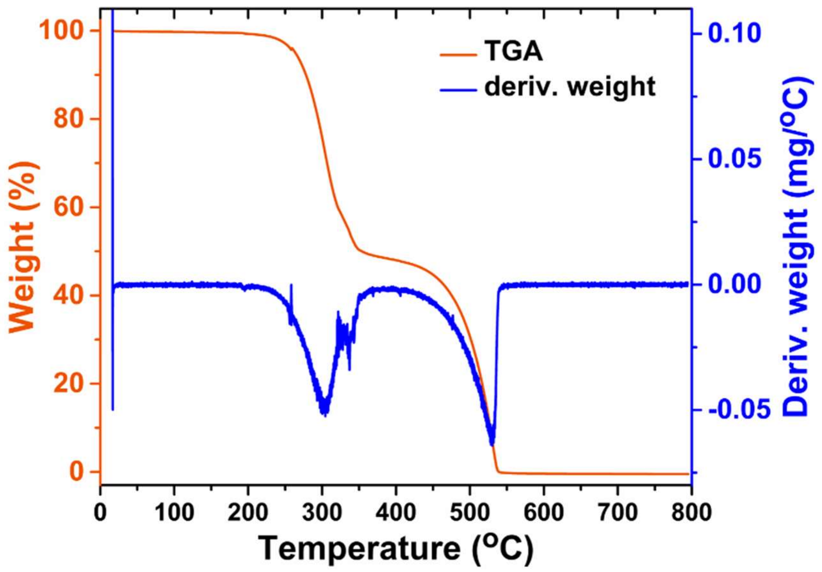

Supplementary Figure 17. TGA curve and corresponding first derivative for the $(PEA)_2PbI_4$ SCM powder.

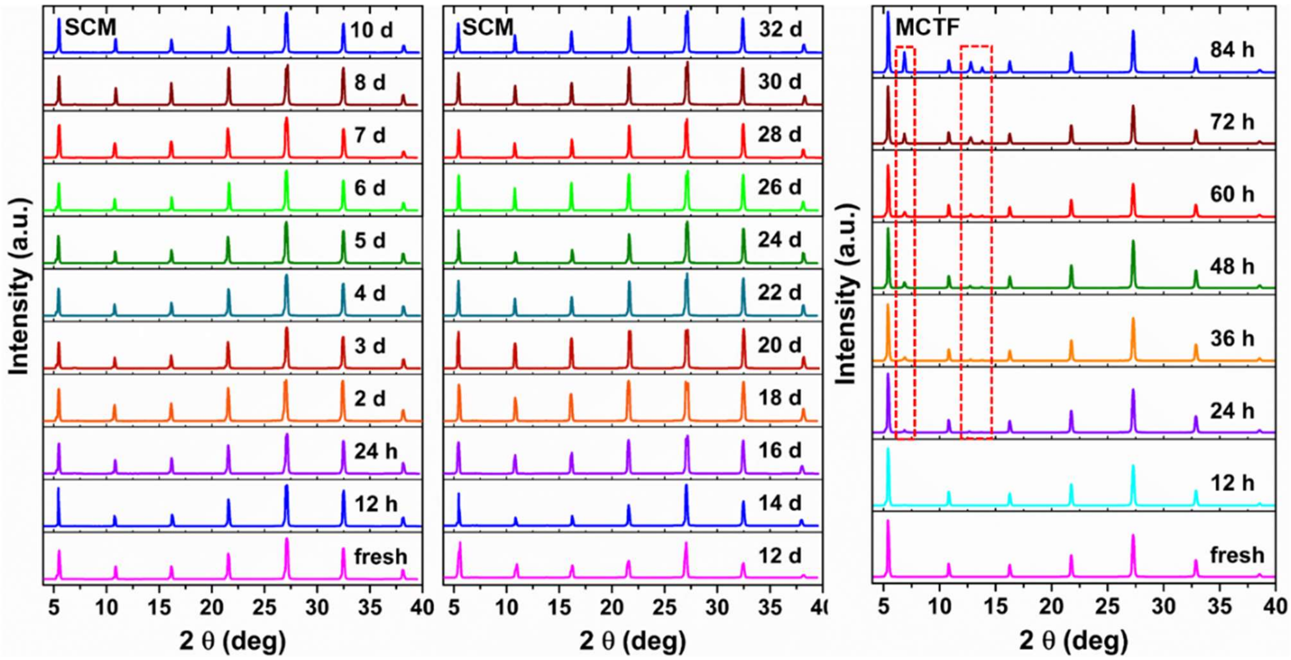

Supplementary Figure 18. The XRD patterns periodically recorded for the $(PEA)_2PbI_4$ SCM and MCTF, stored in high humidity environment with ~65 % relative humidity (RH) at 25 ºC without encapsulation.

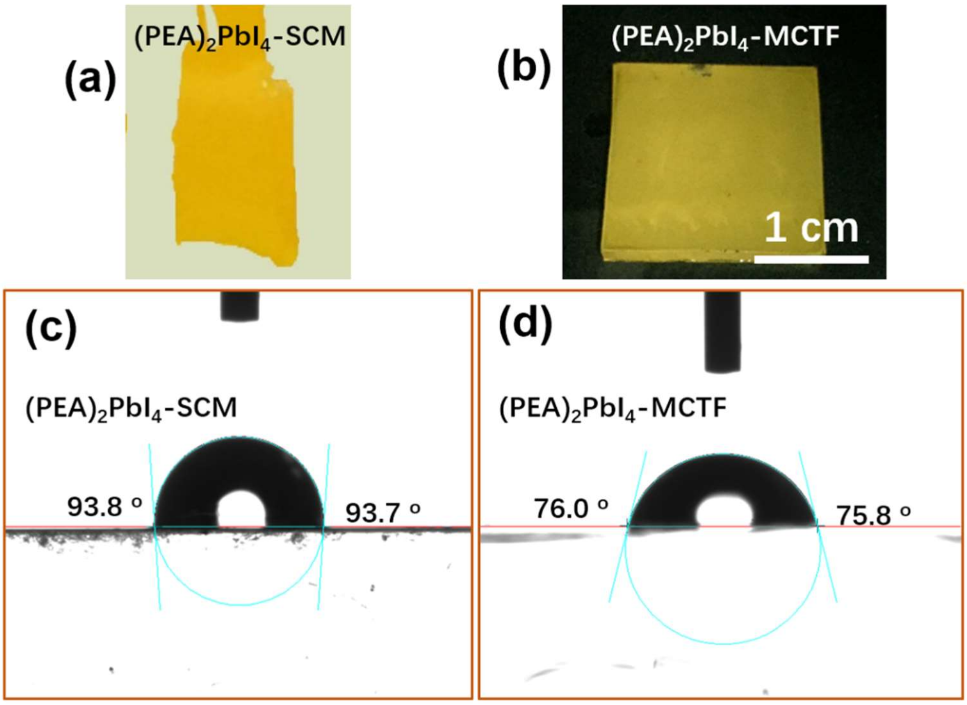

Supplementary Figure 19. Photograph of the $(PEA)_2PbI_4$ SCM (**a**) and $(PEA)_2PbI_4$ MCTF (**b**) used for water contact angle test. Photograph of water droplet on the (**c**) $(PEA)_2PbI_4$ SCM and (**d**) $(PEA)_2PbI_4$ MCTF.

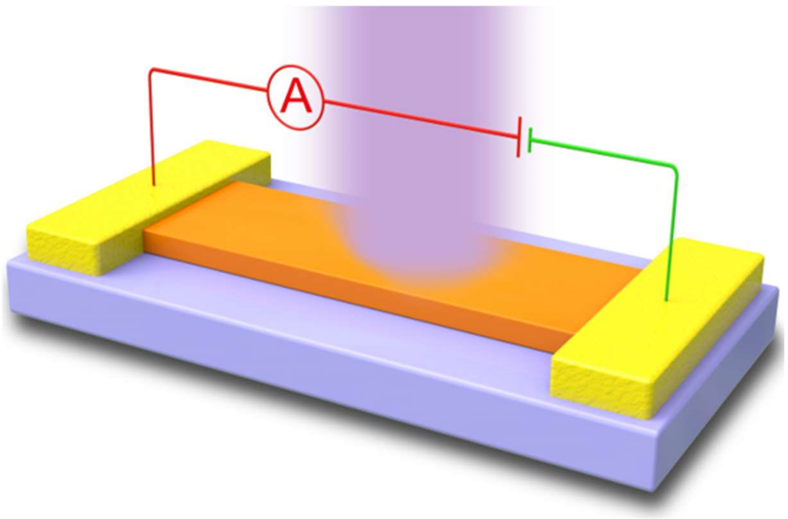

Supplementary Figure 20. Schematic illustration of the photosensor architecture.

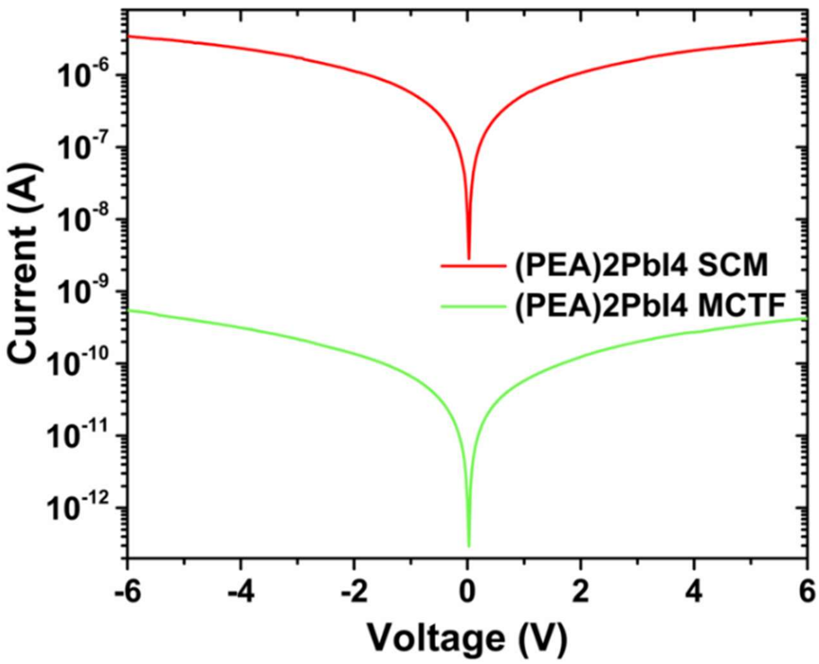

Supplementary Figure 21. Current-voltage (*I-V*) curves of the $(PEA)_2PbI_4$ SCM and $(PEA)_2PbI_4$ MCTF devices measured under 460 nm wavelength illumination with light intensity of 10 mW cm^{-2}.

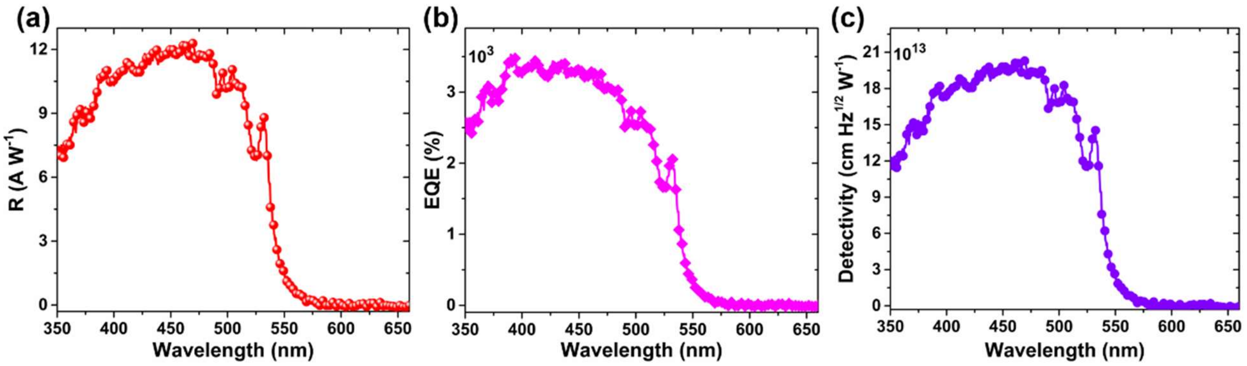

Supplementary Figure 22. (**a**) Responsivity (R) and (**b**) external quantum efficiency (EQE) spectrum of the $(PEA)_2PbI_4$ SCM photosensor measured at a 4 V bias. (**c**) Specific detectivity (D*) calculated from dark noise current of the $(PEA)_2PbI_4$ SCM photosensor.

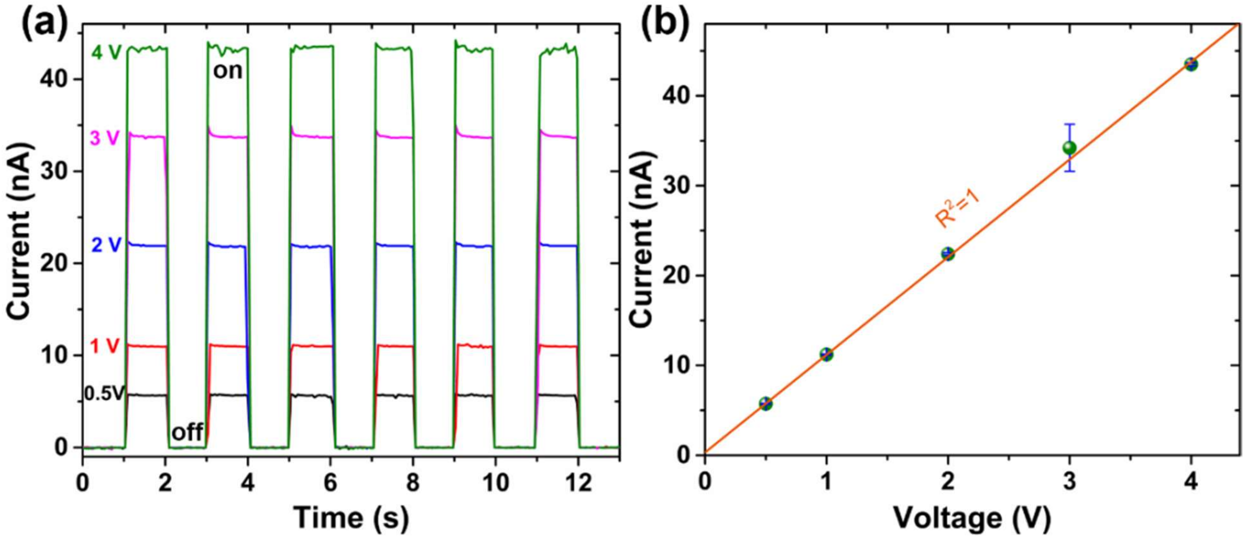

Supplementary Figure 23. (**a**) The time-dependent on/off cycles test of the photocurrent response at a series of bias voltages: 0.5 V, 1 V, 2 V, 3 V, and 4 V under illumination (λ = 460 nm). (**b**) The photocurrent response as a function of bias voltage of the $(PEA)_2PbI_4$ SCM photosensors.

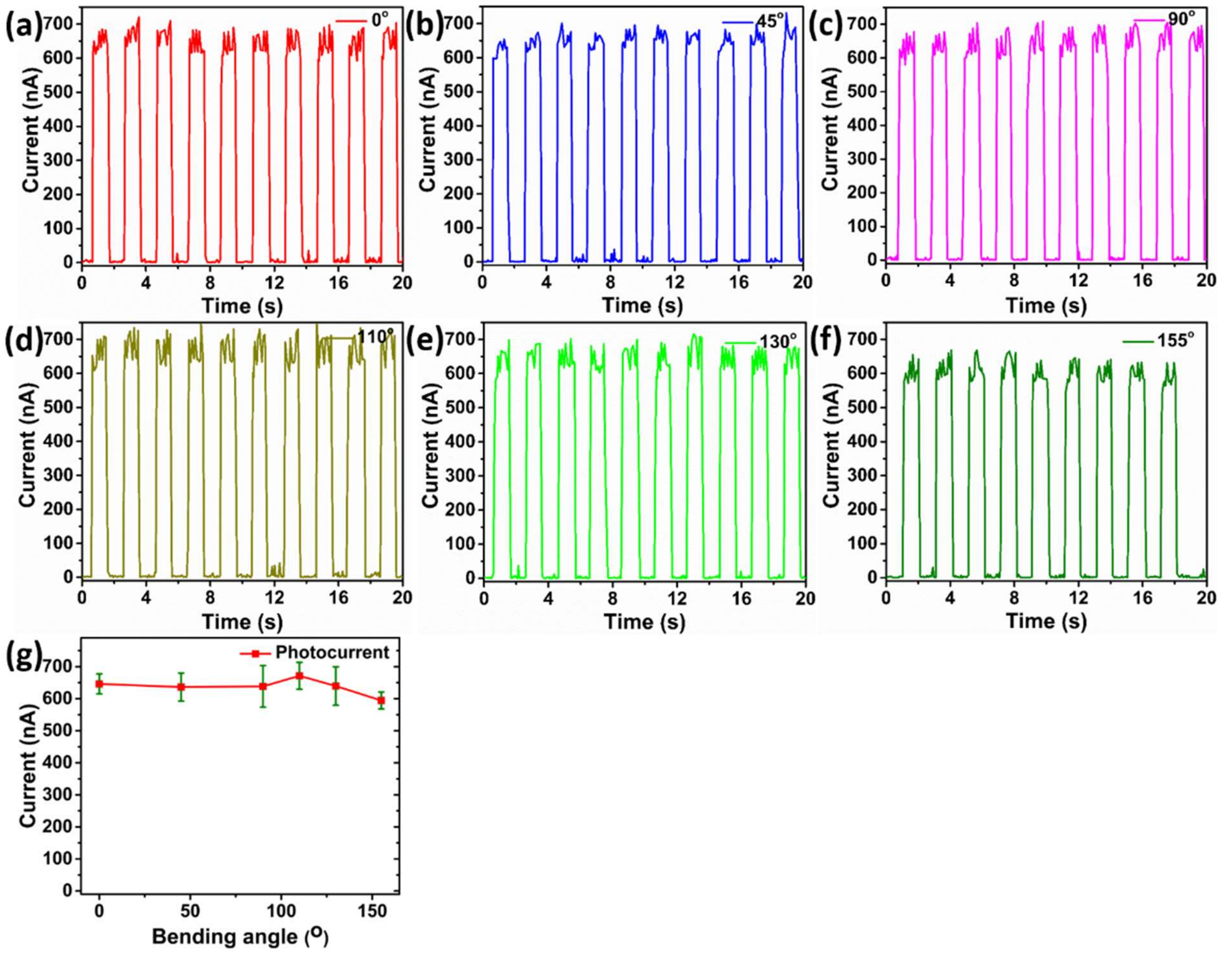

Supplementary Figure 24. (**a-f**) The time-dependent on/off cycles test of the photocurrent response of the flexible planar photosensor at various bending angles. (**g**) The photocurrent response of the flexible planar photosensor bent with various angles from 0° to 155° at a bias voltage of 10 V.

Supplementary Tables.

Supplementary Table 1. The PL peak position, intensity and FWHM values taken from 36 different locations of the large $(PEA)_2PbI_4$ SCM.

Locations	Peak position (nm)	Peak intensity (Counts)	FWHM (nm)	Locations	Peak position (nm)	Peak intensity (Counts)	FWHM (nm)
1	525	38266	15.0	23	525	38697	14.9
2	525	38508	15.0	24	525	38953	14.7
3	525	38656	15.1	25	525	38769	14.8
4	525	38508	15.2	26	525	38697	14.7
5	525	38737	15.1	27	525	38597	14.7
6	525	38656	15.1	28	525	38778	14.9
7	525	38817	15.0	29	525	38795	14.7
8	525	38736	14.9	30	525	39025	14.7
9	525	38427	14.8	31	525	38497	14.8
10	525	38656	14.8	32	525	38739	15.0
11	525	39086	14.7	33	525	38768	14.8
12	525	38575	14.8	34	525	38779	14.7
13	525	38427	14.7	35	525	38794	14.8
14	525	38366	14.7	36	525	38769	14.7
15	525	38868	14.9	37	525	38769	14.7
16	525	38804	14.7	38	525	38859	14.8
17	525	38796	14.7	39	525	38859	14.7
18	525	38954	14.8	40	525	38569	14.9
19	525	38769	14.7	41	525	37998	14.9
20	525	38789	14.7	42	525	38996	15.1
21	525	38964	14.8	43	525	38697	14.8
22	525	38796	14.7	44	525	38773	14.7
Mean	525	38712	14.8				
Standard deviation	525	206.5	0.14				
Minimum	525	37998	14.7				
Median	525	38769	14.8				
Maximum	525	39086	15.2				

Supplementary Table 2. Trap state density of eight devices calculated from *I-V* characteristics for the hole-only devices.

Sample ID	n_{trap} / cm^{-3}
1	8×10^{10}
2	6.95×10^{10}
3	9.42×10^{10}
4	4.32×10^{10}
5	1.32×10^{11}
6	9.81×10^{10}
7	7.34×10^{10}
8	6.37×10^{10}
Mean	8.18×10^{10}
Standard Deviation	2.67×10^{10}
Minimum	4.32×10^{10}
Maximum	1.32×10^{11}

Supplementary Table 3. Trap state density of eight devices calculated from *I-V* characteristics for the electron-only devices.

Sample ID	n_{trap} / cm^{-3}
1	1.14×10^{11}
2	1.32×10^{11}
3	1.45×10^{11}
4	9.89×10^{10}
5	2.45×10^{11}
6	3.42×10^{11}
7	7.48×10^{10}
8	1.77×10^{11}
Mean	1.66×10^{11}
Standard Deviation	0.88×10^{11}
Minimum	0.75×10^{11}
Maximum	3.42×10^{11}

Supplementary Table 4. Dark current of photosensors based on different perovskite single crystals and architectures

Structure	I_{dark} @ V_{Bias}	Reference
Au/3D $MAPbI_3$ (SC)/Au	9.2×10^{-3} mA cm^{-2} at 4 V	1
Au/3D $MAPbI_3$ (SC)/Au	3.7×10^{-2} mA cm^{-2} at 2 V	2
Pt/$MAPbCl_3$ (SC)/Ti/Au	4.15×10^{-4} mA at 15 V	3
Au/$(PEA)_2PbI_4$ (SCM)/Au	3.57×10^{-6} mA cm^{-2} at 2 V	Present work

Supplementary Table 5. Comparative performance parameters of previously reported planar-type perovskite photodetectors and the present $(PEA)_2PbI_4$ SCM device.

Device structure	Responsivity (A/W) @ V_{bias}/ light intensity	Detectivity (Jonse) @V_{bias}	Response speed (rise/decay)	Dark current @ V_{bias}	Ref.
Au/$MAPbI_3$ NW/Au	4.95 @ 1 V/2 nW cm^{-2}	2×10^{13}	< 0.1 ms	1×10^{-10} A @1 V	4
Au/$MAPbI_3$ SC/Au	2.55 @ 1 V/ 1 mW cm^{-2}	–	74/58 µs	1.1×10^{-8} nA @ 4 V	1
Au/$MAPbI_3$ MW/Au	13.57 @ -5 V/500 uW cm^{-2}	5.25×10^{12}	80/240 µs		5
ITO/$MAPbI_3$ NW/ITO	3.49 @ 3 V 10 uW cm^{-2}	5.25×10^{12}	~10^5 µs	4.5×10^{-7} A @ 8 V	6
Pt/$MAPbI_3$ NW/Pt	5×10^{-3}		<500 µs		7
Au/$MAPbI_3$ NW/Au	1.3 @30 V	2.5×10^{12}	200/300 µs	2×10^{-9} A @ 2 V	8
Au/$MAPbI_3$ NW/Au	0.1 @10 V 100 uW cm^{-2}	1.02×10^{12}	300/400 µs	7×10^{-11} A @ 10 V	9
Au/$MAPbI_3$ MW/Au	1.2 @10 V/ 0.1 mW cm^{-2}	2.39×10^{12}	< 10 ms	1.1×10^{-9} A @ 10 V	10
Au/graphene/$MAPbI_{3-x}Cl_x$ nanocrystals/graphene/Au	5.6×10^8 @3 V/	2.8×10^{16}	20/445 µs	~3×10^{-11} A @ 3 V	11
Ag/ZnO NWs/Ag	7.5×10^6 @1 V/0.5 nW cm^{-2}	3.3×10^{17}	0.56/0.32 s	2×10^{-11} A @ 1 V	12
Au/$MAPbI_3$ SC/Au	0.0195 @10 V	1.0×10^{11}@ 4.6 V			13
Au/$MAPbI_3$ SC/Au	2.6 @2 V			1×10^{-6} A @ 3 V	2
Au/$MAPbI_3$ SC/Au	7.92 @4 V/ 10 uW cm^{-2}		< 0.2/0.2 s		14
Au/$MAPbI_3$ SC/Au	7.92 @4 V		39/1.9 µs	10^{-7} A @ 4 V	15
Commercial Si	<1 A W^{-1}	5.8×10^{13}			16
Au/$(PEA)_2PbI_4$ (SCM)/Au	98.17 @4 V /0.08 uW cm^{-2}	1.62×10^{15} @ 4 V	64/52 µs	2.6×10^{-12} A @ 4 V	Present work

Supplementary Notes.

Supplementary Note 1.

Elastic flexing deformation model.

According to the elastic flexing deformation model[17], the stretching length (L) along a′b′ is determined as follow,

$$L = a'b' - ab = \frac{\pi(r+d)\alpha}{180^o} - \frac{\pi r \alpha}{180^o} = \frac{\pi d \alpha}{180^o} \qquad \text{(S1)}$$

Then the flexing angle can be obtained as:

$$\alpha = \frac{180^o L}{\pi d} \qquad \text{(S2)}$$

Where d is half the thickness of the $(PEA)_2PbI_4$ SCM, r is the radius of curvature, α is the flexing angle (**Supplementary Figure 6**).

Supplementary Note 2.

Ultraviolet Photoelectron Spectroscopy (UPS).

UPS is used to determine the Fermi level (E_F) and the valence band maximum (E_V) with respect to vacuum level (E_{VAC}) of the $(PEA)_2PbI_4$ SCM and $(PEA)_2PbI_4$ MCTF[18].

The basic principles and an example spectrum are shown in Supplementary Figure 12. For a photoelectron to escape the sample surface and to be collected, it has to have sufficient energy to overcome the sum of the binding energy (with respect to E_F) of its initial level and the work function Φ, $\Phi = E_{VAC} - E_F$. The work function Φ is determined by the difference between the incident photon energy (21.2 eV) and the binding energy of the secondary electron cut-off (high binding energy edge). In the example spectrum (**Supplementary Figure 12b**), the cut-off binding energy is 16.03 eV as determined by the intersection of the linear portion of the spectrum and the baseline. The work function of this sample is thus Φ = 21.2 - 16.03 = 5.17 eV; that is, E_F is -5.17 eV with respect to E_{VAC}. The difference between E_F and E_V is determined by the intersection of the linear portion of the spectra near the Fermi edge (low binding energy region) with the baseline. The example spectrum (**Supplementary Figure 12b**) has a E_F - E_V = 0.73 eV. Therefore, its valence band maximum E_V = -5.17 -0.73 = -5.9 eV with respect to E_{VAC}. The conduction band minimum (E_C) is further calculated

(**Supplementary Figure 12c**) by adding the optical bandgap, as determined by the position of the lowest exciton absorption peak, to E_V.

Supplementary Note 3

Characterization of the dielectric constant of $(PEA)_2PbI_4$ SCM.

The relative dielectric constants (ε) of the $(PEA)_2PbI_4$ SCMs were estimated from the capacitance-frequency measurement (**Supplementary Figure 15a**). Capacitances (c) of the $(PEA)_2PbI_4$ SCMs were determined from the capacitance-frequency curves and the ε value of $(PEA)_2PbI_4$ SCMs was calculated using equation[19]:

$$\varepsilon = \frac{cd}{\varepsilon_0 A} \qquad \text{(S3)}$$

where d and A are the thickness and the area of the $(PEA)_2PbI_4$ SCMs, and ε_0 is the vacuum permittivity. The ε value of the $(PEA)_2PbI_4$ SCMs can be generally estimated to be 5.8 ± 0.1 (**Supplementary Figure 15b**).

Supplementary Note 4

Characterization of the trap density of $(PEA)_2PbI_4$ SCM.

The trap state density of the $(PEA)_2PbI_4$ SCM was evaluated using the space charge limited current (SCLC) method. Typically, a SCLC *I-V* curve shows two different regimes depending on applied voltages, including transition from ohmic regime ($I \sim V$) at low voltages to SCLC regime ($I \sim V^2$) at high voltages. From this *I-V* curve, the trap density n_{trap} was calculated using the following relation[20]:

$$n_{\text{trap}} = \frac{2V_{\text{TFL}}\varepsilon\varepsilon_0}{eL^2} \qquad \text{(S4)}$$

Where V_{TFL} is the trap-filled limit voltage, L is the thickness of the crystal, ε_0 is the vacuum permittivity, ε is the relative dielectric constant and e is the electron charge.

Supplementary Note 5

Calculation of figures of merit of $(PEA)_2PbI_4$ SCM photosensor.

To evaluate photoresponse characteristics, several parameters need to be determined. Although certain applications require different features, the key figure-of-merit parameters in photodetectors are the responsivity (R), external quantum efficiency (EQE) and specific detectivity (D*).

The R is the ratio of photocurrent to incident light intensity, a signature of how effectively the detector responds to an optical signal[1,2]:

$$\mathrm{R} = \frac{I_\mathrm{p} - I_\mathrm{d}}{A \cdot L_\mathrm{light}} \qquad \text{(S5)}$$

where I_p is the photocurrent, I_d the dark current, A the active area of the device, and L_light is the incident light intensity.

The EQE indicates the photon-electron conversion effciency and is calculated as following[2]:

$$\mathrm{EQE} = \mathrm{R}\frac{hc}{\lambda e} \qquad \text{(S6)}$$

where R is the responsivity, h the Planck constant, c the velocity of light, e the elementary charge (1.6×10^{-19} Coulomb), and λ the wavelength of incident light.

The D*, which representing the minimum signal that can be detected of a photodetector. To obtain the D*, we first directly recorded the noise current by a dynamic signal analyzer, a method that has been well established by Tang et al. and Huang et al[21,22]. Based on the measured dark current noise (i_n) and the R of the devices, the noise equivalent power (NEP) of the device can be calculated according to the equation:

$$\mathrm{NEP} = \frac{i_\mathrm{n}}{R} \qquad \text{(S7)}$$

Thus, we can obtain the specific detectivity (D*) by the following equation:

$$D^* = \frac{\sqrt{AB}}{\mathrm{NEP}} = \frac{\mathrm{R}\sqrt{AB}}{i_\mathrm{n}} \qquad \text{(S8)}$$

where B the electrical bandwidth, NEP is the amount of light equivalent to the noise level of a device and i_n is the noise current under the same conditions. A noise is associated with current fluctuation and could be directly calculated from the dark current. If the shot noise from the dark current (J_d) is the major contribution of the overall photodetector noise, D* can be simplified as[3]:

$$D^* = \frac{\mathrm{R}\sqrt{A}}{\sqrt{2eI_\mathrm{d}}} \qquad \text{(S9)}$$

where e is the absolute value of electron charge (1.6×10^{-19} Coulomb), and I_d the dark current density.

Supplementary References:

1. Lian, Z. *et al.* High-Performance Planar-Type Photodetector on (100) Facet of $MAPbI_3$ Single Crystal. *Sci. Rep.* **5**, 16563 (2015).
2. Liu, Y. *et al.* Thinness- and Shape-Controlled Growth for Ultrathin Single-Crystalline Perovskite Wafers for Mass Production of Superior Photoelectronic Devices. *Adv. Mater.* **28**, 9204-9209 (2016).
3. Maculan, G. *et al.* $CH_3NH_3PbCl_3$ Single Crystals: Inverse Temperature Crystallization and Visible-Blind UV-Photodetector. *J. Phys. Chem. Lett.* **6**, 3781-3786 (2015).
4. Gao, L. *et al.* Passivated Single-Crystalline $CH_3NH_3PbI_3$ Nanowire Photodetector with High Detectivity and Polarization Sensitivity. *Nano Lett.* **16**, 7446-7454 (2016)
5. Deng, W. *et al.* Aligned Single-Crystalline Perovskite Microwire Arrays for High-Performance Flexible Image Sensors with Long-Term Stability. *Adv. Mater.* **28**, 2201-2208 (2016).
6. Hu, X. *et al.* High-Performance Flexible Broadband Photodetector Based on Organolead Halide Perovskite. *Adv. Funct. Mater.* **24**, 7373-7380 (2014).
7. Horvath, E. *et al.* Nanowires of methylammonium lead iodide ($CH_3NH_3PbI_3$) prepared by low temperature solution-mediated crystallization. *Nano Lett.* **14**, 6761-6766 (2014).
8. Deng, H. *et al.* Growth, patterning and alignment of organolead iodide perovskite nanowires for optoelectronic devices. *Nanoscale* **7**, 4163-4170 (2015).
9. Deng, H. *et al.* Flexible and Semitransparent Organolead Triiodide Perovskite Network Photodetector Arrays with High Stability. *Nano Lett.* **15**, 7963-7969 (2015).
10. Liu, Y. *et al.* Inkjet-Printed Photodetector Arrays Based on Hybrid Perovskite $CH_3NH_3PbI_3$ Microwires. *ACS Appl. Mater. Interfaces* **9**, 11662-11668 (2017).
11. Lin, Y., Lin, G., Sun, B., Guo, X. Nanocrystalline Perovskite Hybrid Photodetectors with High Performance in Almost Every Figure of Merit. *Adv. Funct. Mater.* **28**, 1705589 (2018).
12. Liu, X., Gu, L., Zhang, Q., Wu, J., Long, Y., Fan, Z. All-printable band-edge modulated ZnO nanowire photodetectors with ultra-high detectivity. *Nat. Commun.* **5**, 4007 (2014).
13. Ding, J. *et al.* Design Growth of $MAPbI_3$ Single Crystal with (220) Facets Exposed and Its Superior Optoelectronic Properties. *J. Phys. Chem. Lett.* **9**, 216-221 (2018).
14. Fang, H.J. *et al.* A self-powered organolead halide perovskite single crystal photodetector driven by a DVD-based triboelectric nanogenerator. *J. Mater. Chem. C* **4**, 630-636 (2016).
15. Zhang, Y., Liu, Y., Yang, Z., Liu, S.Z. High-quality perovskite $MAPbI_3$ single crystals for broad-spectrum and rapid response integrate photodetector. *J. Energy Chem.* **27**, 722-727 (2018).
16. Guo, X. *et al.* High-Responsivity Si Photodiodes at 1060 nm in Standard CMOS Technology. *IEEE Electron Device Letters* **39**, 228-231 (2018).

17. Zhang, N.H., Xing, J.J. An alternative model for elastic bending deformation of multilayered beams. *J. Appl. Phys.* **100**, 103519 (2006).
18. Chuang, C.H., Brown, P.R., Bulovic, V., Bawendi, M.G. Improved performance and stability in quantum dot solar cells through band alignment engineering. *Nat. Mater.* **13**, 796-801 (2014).
19. Kambale, R.C., Adhate, N.R., Chougule, B.K., Kolekar, Y.D. Magnetic and dielectric properties of mixed spinel Ni-Zn ferrites synthesized by citrate–nitrate combustion method. *J. Alloy Compd.* **491**, 372-377 (2010).
20. Bube, R.H. Trap Density Determination by Space-Charge-Limited Currents. *J. Appl. Phys.* **33**, 1733 (1962).
21. Pan, W. *et al.* $Cs_2AgBiBr_6$ single-crystal X-ray detectors with a low detection limit. *Nat. Photon.* **11**, 726-732 (2017).
22. Fang, Y., Dong, Q., Shao, Y., Yuan, Y., Huang, J. Highly narrowband perovskite single-crystal photodetectors enabled by surface-charge recombination. *Nat. Photon.* **9**, 679-686 (2015).
